# Atomic-Scale Electric Potential Landscape across Molecularly Gated Bilayer MoS_2_ Resolved by Photoemission

**DOI:** 10.1021/acsnano.5c10363

**Published:** 2025-09-08

**Authors:** Laura Scholz, Patrick Amsalem, Lennart Frohloff, Rongbin Wang, Emily Albert, Kan Tang, Stephen Barlow, Seth R. Marder, Norbert Koch

**Affiliations:** † Insitut für Physik and Center for the Science of Materials Berlin, Humboldt-Universität zu Berlin, Berlin 12489, Germany; ‡ Helmholtz-Zentrum Berlin für Materialien und Energie GmbH, 12489 Berlin, Germany; § Renewable and Sustainable Energy Institute (RASEI), University of Colorado Boulder, Boulder, Colorado 80309, United States; ∥ Department of Chemical and Biological Engineering and Department of Chemistry, University of Colorado Boulder, Boulder, Colorado 80309, United States

**Keywords:** 2D semiconductors, photoemission spectroscopy, Stark shift, quantum confinement, molecular dopants

## Abstract

Electric gating in atomically thin field-effect devices based on transition-metal dichalcogenides has recently been employed to manipulate their excitonic states, even producing exotic phases of matter, such as an excitonic insulator or Bose–Einstein condensate. Here, we mimic the electric gating effect of a bilayer-MoS_2_ on graphite by charge transfer induced by the adsorption of molecular p- and n-type dopants. The electric fields produced are evaluated from the electronic energy-level realignment and Stark splitting determined by X-ray and UV photoelectron spectroscopy measurements and compare very well with literature values obtained by optical spectroscopy for similar systems. We then show that analysis of the inhomogeneous broadening and energy shifts of the quantum-well states of the valence band allows extraction of the full electric potential profile and charge-density redistribution across the entire heterojunction with atomic-scale precision, which is not accessible by other methods.

## Graphical abstract

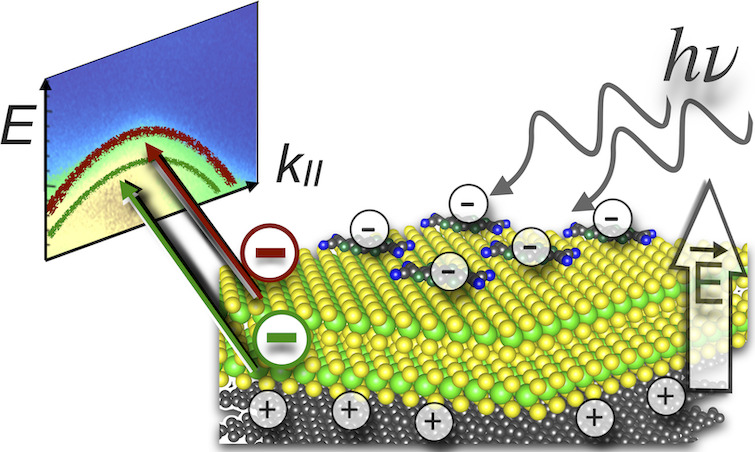

Transition-metal dichalcogenides (TMDCs) are two-dimensional (2D) van der Waals semiconductors with the formula MX_2_ (M being a transition metal and X a chalcogen atom). The 2H phase of MX_2_ (MoS_2_, WS_2_, MoSe_2_, and WSe_2_) shows promise for optoelectronic device implementation due to their direct band gap as well as specific electronic and optical properties.^1–6^ In the monolayer (ML) limit, MoS_2_ has a direct band gap at the K-point of the Brillouin zone (BZ) due to the low dimensionality and broken inversion symmetry.^7–9^ This, together with strong spin–orbit coupling, enables possible pathways toward valley- and spintronic applications.^7^ For homobilayer (BL) TMDC stacks (e.g., BL-MoS_2_), the emergence of low-energy quantum-well (QW) states at the reciprocal lattice point Γ reduces the band gap, turning the material from a direct semiconductor in the ML limit to an indirect one in the BL case (with indirect transition between Γ-Q or Γ-K depending on the layer stacking)^9^ (Figure S1). Also, as the inversion symmetry is restored in BL-TMDCs, features such as valley polarization or valley-Hall effects are lost but can be recovered by applying electric fields perpendicular to the BL-TMDC plane.^10,11^

ML-TMDCs feature quasiparticle many-body states such as excitons, trions, and biexcitons, with the exciton binding energy in the range of ca. 250 meV.^12–14^ The photoluminescence energy of these excitations can be tuned through electric gating and the associated quantum confined Stark effect (QCSE).^15–17^ BL stacks show a set of even more interesting interlayer excitonic phenomena, which can be engineered through different means.^18–25^ For instance, exposing a BL-TMDC to an electric field through gating in field-effect transistor-like devices allows manipulating the interlayer excitons via tuning of the BL band structure through Stark splitting^25,26^ (Figure 1a,b). Controlling the band alignment in these BLs appears as a means to potentially set up the appropriate conditions needed for a variety of more exotic phenomena, ranging from Bose–Einstein condensates to excitonic insulators^27–30^ to emerge, positioning these BLs as key materials for the advancement of heat-loss-free information-transmission devices. Toward these aims, electric gating of a BL-MoS_2_ functionalized with p-dopant molecules was recently reported to produce electric fields with magnitude beyond the dielectric breakdown limit of 0.15 V/nm normally achievable by electric gating.^26,31^ Such investigations permitted the observation of resonant coupling between intra- and interlayer excitons.^18–20,26^ However, all the above studies focused on the excited states of BL-TMDCs, and no experimental work has so far elucidated the underlying ground-state electronic structure under an electric field, the knowledge of which is a prerequisite for engineering these complex heterostructures toward desired functionality.

**Figure 1:**
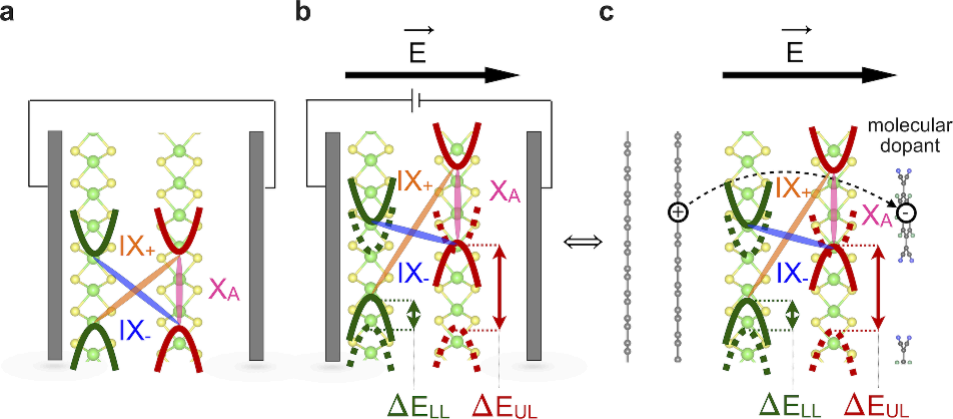
Energy-level alignment in BL-MoS_2_ at the K-point (a) without and (b) with an applied electric field through direct gating. (c) Schematic example of an electric field induced by molecular adsorption of a molecular electron acceptor. The solid black arrow shows the orientation of the electric field in the analogous cases of (b) and (c). Curved parabolas show the local valence-band maximum (VBM) and conduction-band minimum (CBM) around reciprocal-lattice point K for the lower layer (LL) of MoS_2_ (green) and for the upper layer of (UL) MoS_2_ (red). The dashed parabolas in (b) and (c) show the position of the CBM and VBM without an electric field, as in (a). Δ*E*_LL_ and Δ*E*_UL_ represent the energy shifts experienced by the energy levels of the LL and UL MoS_2_, respectively, in an electric field. The pink, orange, and purple ellipses show the X_A_ intralayer and the two interlayer (IX_+_, IX_–_) excitonic transitions, respectively. In (c), the dashed arrow shows the charge transfer (CT) occurring upon molecular electron-acceptor deposition, showing the formation of holes (+) in the substrate region and electrons (−) in the molecular layer.

Electric fields can emerge via the adsorption of small molecules or atoms. The effect of p-dopant molecules on the band alignment and doping of TMDCs has been addressed for ML-MoS_2_. When using highly oriented pyrolytic graphite (HOPG) as a substrate, Stark shifts were observed to result from a HOPG-to-molecule charge transfer (CT).^32,33^ The TMDC was found to behave as a “bridge” dielectric placed between the plates of a capacitor (the bottom HOPG and the top molecular layer as plates). Possible Stark shifts occurring upon n-doping at the surface of alkali-doped multilayer TMDCs were reported through monitoring the modulation of the valence and conduction band extrema by angle-resolved photoemission spectroscopy (ARPES).^34^ The transition from indirect-to-direct band gap may be achieved at sufficiently high fields, even for multilayer samples. Still, the impact of the adsorption of p- and n-dopant molecules on the electronic structure of BL-TMDCs has not been addressed so far, likely linked to experimental difficulties associated with the use of normally macroscopic measurement techniques (like ARPES and X-ray photoemission spectroscopy (XPS)) applied to systems such as BL-TMDC that are usually achieved on micrometer scales.^35–38^

Here, we overcome the experimental challenges of combining ARPES/XPS on BL-MoS_2_ by fabricating centimeter-scale samples with over 95% coverage of BL-MoS_2_ on a HOPG substrate (detailed information regarding the coverage estimation is provided in the SI in Section S1b, Figures S2–S15). We can now fill the knowledge gap related to the ground-state electronic structure, i.e., the Stark shifts experienced by the electronic valence and core levels of a BL-MoS_2_ when tunable electric fields are applied through molecular gating, i.e., through the adsorption of organic dopants as depicted in Figure 1c. Such BL-MoS_2_ does not exhibit a defined twist angle, as each of the individual MLs consists of azimuthally randomly rotated domains, but nevertheless features the expected band structure and indirect band gap related to the presence of QW states at the Γ point of the BZ.^9,39^ We determine the energy-level realignment experienced by the BL-MoS_2_ upon molecular gating through the deposition of 1,3,4,5,7,8-hexafluoro-tetracyano-naphthoquinodimethane (F_6_TCNNQ) and ruthenium pentamethylcyclopentadienyl mesitylene dimer ([RuCp*mes]_2_), prototypical molecular p- and n-dopants, respectively (Figures S16 and S17). F_6_TCNNQ is one of the strongest electron-acceptor molecules available with an electron affinity (EA) amounting to ca. 5.6 eV.^40^ [RuCp*mes]_2_ has a strong electron-donor character due to its low estimated effective ionization energy (IE) of ca. 2.8 eV, rendering it a superior n-type dopant.^41,42^ [RuCp*mes]_2_ consists of two monomers bound by a strong central carbon bond. When adsorbed on surfaces, this bond undergoes cleavage upon loss of an electron, which results in the formation of a molecular monomeric cation, RuCp*mes^+^, on the surface, while the other neutral monomer can transfer a second electron to the surface or possibly desorb.^42^

In line with previous reports using ML-MoS_2_,^32,43^ molecular p-doping of BL-MoS_2_ with F_6_TCNNQ results in a HOPG-to-molecule electron transfer, leading to electric fields and giant Stark splitting up to 0.4 V·nm^–1^ and 0.2 eV, respectively.^26^ In contrast, n-doping of BL-MoS_2_ by [RuCp*mes]_2_ shows a more complex charge redistribution; in addition to a potential drop between the substrate and the molecular overlayer, the emergence of a partially filled conduction band reveals a large carrier concentration in the BL-MoS_2_.

The electric potential profile within BL-MoS_2_ is first assessed by XPS and then linked to the Mo-derived valence-band structure at the K-point. Additionally, we propose that the evolution of the line shape of the energy-distribution curves (EDC) at the Γ-point intimately relates to the details of the potential landscape in the direction normal to the MoS_2_ basal plane. Accordingly, we then simulate the effect of the potential gradient on the EDC at Γ and reconstruct a detailed potential profile, thereby providing insights into the charge density redistribution across the heterojunctions.

Finally, the impact of the electric fields on the two QW states at Γ is addressed by numerically modeling a potential well modified with the determined potential profile. The excellent agreement of this fundamental approach with the experimental results allows us to gain comprehensive knowledge of the charge rearrangement—with experimentally unprecedented spatial resolution on the atomic scale—leading to the energy level realignment and formation of interface dipoles at these TMDC-based van der Waals heterojunctions.

## Results

### Sample Work Function Upon Adsorption of p- and n-Type Molecular Dopants

The observed dependence of the secondary-electron cutoff spectra on the nominal thickness of the molecular layers, from which the work function (WF) and its changes (ΔWF) are deduced, are shown in Figure 2 for the deposition of the acceptor F_6_TCNNQ and donor [RuCp*mes]_2_. The clean BL-MoS_2_ WF is about 4.7 eV, the same as that of bare HOPG, in line with the expected vacuum level alignment at this van der Waals interface. ΔWF values of up to +0.95 and −2.5 eV are found for the deposition of F_6_TCNNQ and [RuCp*mes]_2_, respectively. These trends are consistent with electron transfer *toward* F_6_TCNNQ and *from* [RuCp*mes]_2_. ΔWF takes place almost entirely within the first 3 Å of molecular layer thickness, indicating that charge transfer predominantly involves the first molecular layer. The final WF values obtained intrinsically relate to the IE and EA of the molecules and are in line with previous reports.^44^ At this point, one might be tempted to conclude that the electric field across the BL-MoS_2_ (i.e., between HOPG and the molecular layer) simply equals ΔWF/*e·d,* with *e* being the electron charge and *d* being the distance between the positive and negative poles resulting from the charge transfer. However, as shown in the following, this assumption is entirely inadequate.

**Figure 2:**
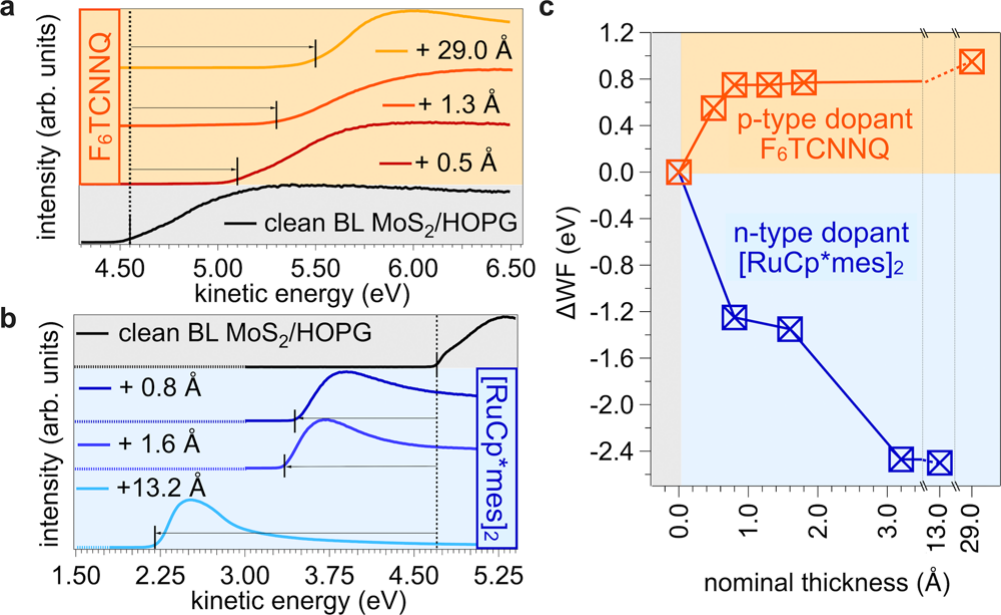
Work function (WF) evolution for different molecular charge dopant depositions. (a, b) Secondary-electron cutoff spectra (SECO) and (c) nominal thickness-dependent work-function change (ΔWF) evolution upon F_6_TCNNQ and [RuCp*mes]_2_ deposition.

### Stark Shifts from XPS Core Level Analysis

We first describe the changes in the BL-MoS_2_ core levels by the representative sub-ML deposition of 0.5 Å F_6_TCNNQ and 1.6 Å [RuCp*mes]_2_. For such low molecular coverages, the MoS_2_ core levels can be clearly observed using XPS, and even their valence bands can be seen in ARPES (discussed below), enabling a comprehensive analysis of the electronic properties. Yet, already large ΔWF values of +0.55 eV for F_6_TCNNQ and −1.3 eV for [RuCp*mes]_2_ are present for these low coverages.

#### F_6_TCNNQ

The Mo 3d and S 2p core levels of BL-MoS_2_ before and after F_6_TCNNQ deposition are shown in Figure 3a,b. Because the EA of F_6_TCNNQ is ca. 1 eV lower than the IE of MoS_2_, the CT is expected to occur between HOPG and the molecules, thus shifting the energy levels of each MoS_2_ differently, because the lower layer (LL, contacting the HOPG) and the upper layer (UL, contacting the molecular layer) reside at different electric potentials.^45^ This can be monitored via the binding energy (BE) shift of the core levels. The core levels of clean BL-MoS_2_ can be fitted with one component, as expected because of the van der Waals nature of the stack and the absence of an electric field. After F_6_TCNNQ deposition, both the Mo 3d and S 2p core levels broadened by ca. 20% and their peak maximum shifted by ca. 170 meV to lower BE. The two components representative of LL and UL cannot be visually resolved due to the finite experimental resolution and natural lifetime broadening. Angle-dependent XPS measurements taken at 0° photoelectron emission (along the surface normal; more bulk sensitive) and 70° emission (more surface sensitive) enhance either the LL or UL spectral component, according to the short electron inelastic mean free path (λ) of ca. 28 Å for the studied kinetic energy range (Figure S18). A shift of the overall spectral envelope of 60 meV BE between the two emission angles is observed for both the Mo 3d and S 2p core levels, as clearly revealed by the difference spectra (Figure 3a). This is due to the enhancement of the near-surface UL, which sits at a higher potential than the bottom LL. We therefore fit the core levels after F_6_TCNNQ deposition with a two-component model to represent LL and UL. In this fitting procedure, the area ratio was constrained according to the attenuation factor given by the λ of 28 Å. This gives an intensity ratio of the bottom-to-surface components *I*_LL_ */I*_UL_ at 0 and 70° emission angles of 0.78 and 0.49, respectively. The peak BE, width, and shape of the employed Voigt functions were free parameters but forced to remain constant for the two angles (further details are provided in the SI, Section S5). The result of this constrained fitting yields two components, corresponding to the LL and UL, for both Mo 3d and S 2p. Compared to the pristine BL-MoS_2_, broadening of the LL and UL components by about 15–20% is found. The LL levels are observed to shift by (90 ± 30) meV and the UL levels by (250 ± 30) meV to lower BE. Comparing the 250 meV shift of UL with the ΔWF of 550 meV implies that an interface dipole of ca. 300 meV is present at the F_6_TCNNQ/UL-MoS_2_ interface, providing an initial assessment of the surface-normal potential landscape (Figure 3c,d). The same fitting procedure was applied consistently to spectra taken at higher F_6_TCNNQ coverages (Figure S21).

**Figure 3:**
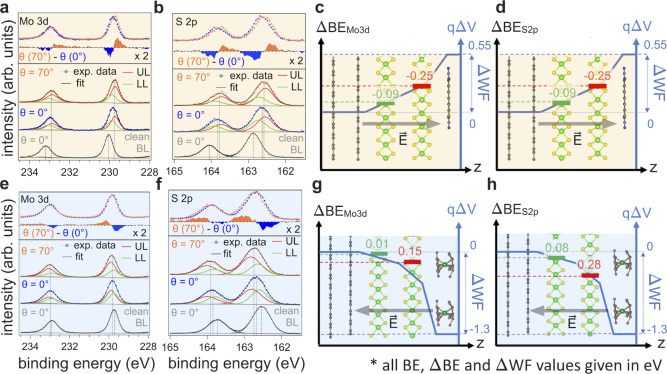
Angle-dependent XPS analysis and Stark-splitting extraction for the initial electric potential landscape assessment in BL-MoS_2_ upon deposition of 0.5 Å F_6_TCNNQ and 1.6 Å [RuCp*mes]_2_. XPS spectra of the Mo 3d and S 2p core levels for clean BL-MoS_2_ and after deposition of (a, b) 0.5 Å F_6_TCNNQ and (e, f) 1.6 Å [RuCp*mes]_2_. The blue and orange spectra were measured at 0 and 70° photoelectron emission angles. Their difference spectrum reveals an angle-dependent energy shift due to UL (upper MoS_2_ layer) residing at a different potential than LL (lower MoS_2_ layer). The 0 and 70° emission angle spectra are fitted with two components (green curve for LL and red curve for UL). The change in electric potential along the *z*-direction (surface normal) as deduced from the Mo 3d and S 2p core levels for 0.5 Å F_6_TCNNQ is shown in (c, d) and for the 1.6 Å [RuCp*mes]_2_ in (g, h). Note the different shifts of Mo 3d and S 2p core levels for 1.6 Å [RuCp*mes]_2_ due to the reduction of Mo because of strong n-doping of the BL-MoS_2_.

#### [RuCp*mes]_2_

The same set of angle-dependent XPS measurements was performed for the deposition of donor [RuCp*mes]_2_. As the potential is now decreasing toward the surface because of a reversal of the CT direction compared to the acceptor, the MoS_2_ core levels shift to higher BE. The global S 2p peak shifts by ca. 150 meV, while the Mo 3d peak shifts only by ca. 100 meV, and both core levels are found ca. 40% broadened, which we anticipate is due to the differential shifts of LL and UL. We again fit the core level spectra with the two-component model and determine a (280 ± 30) meV and (80 ± 30) meV shift for LL and UL from the S 2p spectra, respectively. The magnitude of the Mo 3d LL and UL shifts is found to be much smaller, amounting to (150 ± 30) meV and (10 ± 30) meV only. As evidenced below by ARPES, the difference between the S 2p and Mo 3d shifts originates from the reduction of the effective molybdenum oxidation state upon electron transfer from the [RuCp*mes]_2_ molecules to the MoS_2_; the bottom conduction band has a predominant contribution from Mo *d*_*z* ^2^_ orbitals, with essentially no contribution from sulfur orbitals, and therefore the effective sulfur oxidation state is little affected by reduction of MoS_2_. Consequently, only the BE shifts determined for the S 2p core levels should be used as reliable markers for assessing the change in the electric potential of each layer. The difference between ΔWF and the S 2p energy shift allows us to conclude on the formation of an interface dipole of ca. 1.00 eV at the [RuCp*mes]_2_/UL interface, as depicted in Figure 3g,h. Consistent results were observed for other deposition thicknesses of the electron donor (Figure S22).

### Valence Energy Levels from ARPES

#### Clean BL-MoS_2_

Figure 4a,b shows the energy-momentum ARPES maps, curvature representations, and corresponding EDCs of the clean BL-MoS_2_ on HOPG at the Γ- and K-points. Although no specific azimuthal angles were chosen for the measurements, the ARPES spectra show sharp band dispersion, consistent with that expected along the Γ-K direction. The lack of sensitivity to the azimuthal angle relates to the structural azimuthal disorder of the MoS_2_ layer—analogous to a 2D powder—and the large density of states (DOS) located along the high symmetry directions (Γ-K and Γ-M) of the BZ.^39^ Around the Γ-point, the top valence band consists of two bands Γ_1_ and Γ_2_, centered at 1.61 and 2.10 eV BE, respectively, as seen in Figure 4a. These arise from the quantum confinement and hybridization of the out-of-plane Mo *d*_*z* ^2^_ and S *p*_*z*_ orbitals, allowing us to identify the number of layers being probed.^9^ We note that the relative intensity of these two bands is strongly photon-energy-dependent (not shown) and cannot be used to estimate the bilayer coverage. At the K-point, the two in-plane Mo *d*_*x* ^2^–*y* ^2^,*xy*_-derived bands around 1.8 eV BE are split by ca. 170 meV because of spin–orbit-coupling, with the valence band maximum (VBM) at (1.70 ± 0.03) eV.^46^ As a result, the BL-VBM is at the Γ-point, which is in agreement with theoretical expectations. Furthermore, as the quasiparticle band gap is in the range of 2 eV,^47^ the Fermi level (*E*_F_) is closer to the conduction band minimum (CBM) and BL-MoS_2_ exhibits slight apparent n-type character.

**Figure 4:**
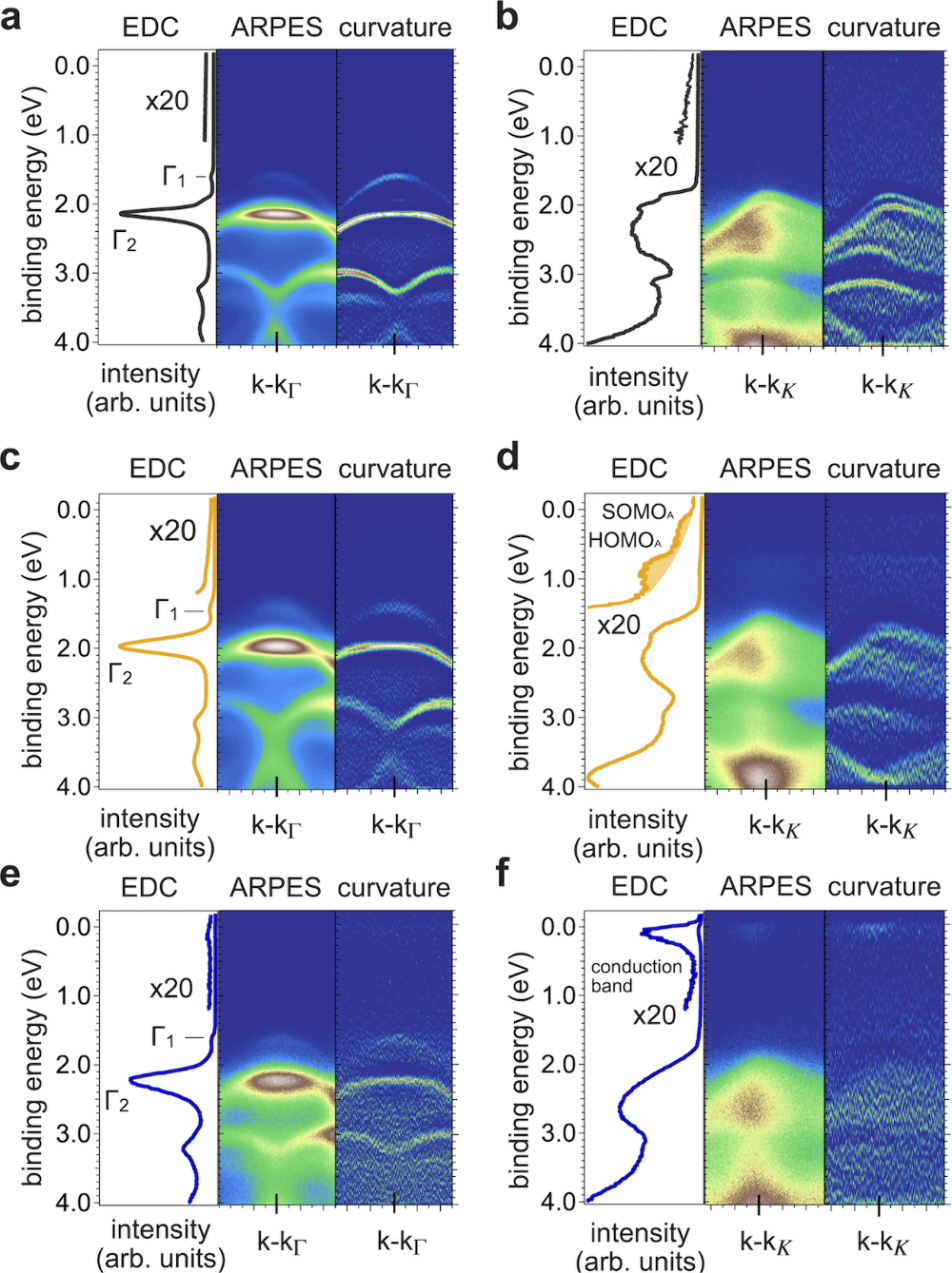
Energy-momentum ARPES maps of BL-MoS_2_ before and after molecular deposition. Reciprocal-lattice point Γ of (a) clean BL-MoS_2_, and upon deposition of (c) 0.5 Å F_6_TCNNQ and (e) 1.6 Å [RuCp*mes]_2_. Energy-momentum ARPES maps at K of (b) clean BL-MoS_2_, (d) 0.5 Å F_6_TCNNQ, and (f) 1.6 Å [RuCp*mes]_2_. Each graph shows the EDC at Γ or K (±1°), the as-measured ARPES map, and the corresponding curvature representation.

#### F_6_TCNNQ

Figure 4c,d shows the ARPES maps, curvatures, and EDCs of 0.5 Å F_6_TCNNQ/BL-MoS_2_ at the Γ- and K-points. Upon the deposition of F_6_TCNNQ, the formation of molecular anions due to electron transfer is observed via the emergence of two DOS features near E_F_ at the K-point (Figure 4d). These features are attributed to the anion’s singly occupied molecular orbital (SOMO_A_; derived from the lowest unoccupied molecular orbital of the neutral molecule) and relaxed highest occupied molecular orbital (HOMO_A_).^33^ The observation of these molecular states solely at higher parallel momentum (*k*_*//*_) is in line with the expected dependency of the wavevector *k* of these molecular orbitals usually reported for flat-lying π-conjugated organic molecules on surfaces.^48–50^ A general shift of the MoS_2_ features toward lower BE is observed, consistent with the electric field resulting from the HOPG-to-molecule electron transfer, and also in line with the XPS results. This shift amounts to about 175 meV for the most intense valence feature at Γ_2_ and about 250 meV for the VBM at the K-point. In addition, a global broadening of the features is observed, which is striking for Γ_2_ as its full-width at half-maximum increases by 55%, going from ca. 140 to 220 meV, and the spin–orbit coupling at the K-point also appears less resolved. This broadening may be due to momentum scattering of the photoelectrons by the molecular layer as previously reported;^51–53^ but as explained further below, we relate it predominantly to the electric potential gradient within the BL-MoS_2_.^54,55^ In analogy to the XPS analysis, we simulate the changes in the valence region at the K-point, where the excitonic transitions take place in optical studies. At the K-point, the VBM consists of in-plane Mo *d*_*x* ^2^–*y* ^2^,*xy*_ orbitals and the bands due to LL and UL should therefore be affected by the electric fields in the same manner as the Mo core levels. To verify this, we proceed with momentum integration of the ARPES map around the K-point from Figure 4b,d and obtain a momentum-integrated EDC for the clean and F_6_TCNNQ-covered BL-MoS_2_. Comparing these momentum-integrated EDCs allows us to exclude the possible influence of the MoS_2_ photoelectron scattering by the molecular layer in the ARPES map.^51–53^ We then duplicate the EDCs of the clean BL-MoS_2_ and shift them in energy by the amount determined by XPS for LL and UL, as the shifts are of electric origin. We rescaled the spectral intensity to account for the relative attenuation of the LL/UL spectral weight (with ca. 70% of the signal coming from the top layer, yielding a λ of ca. 1.4 ML-MoS_2_). As shown in Figure 5a, summing up the shifted LL and UL contributions allows close reproduction of the experimental EDC of the F_6_TCNNQ/BL-MoS_2_ sample around the K-point. This enables us to assess with high confidence the realignment of the valence levels at the K-point as depicted in Figure 5b, which is the key quantity determining the interlayer exciton energies observed by optical measurements of gated BL-TMDC systems.^26^

**Figure 5:**
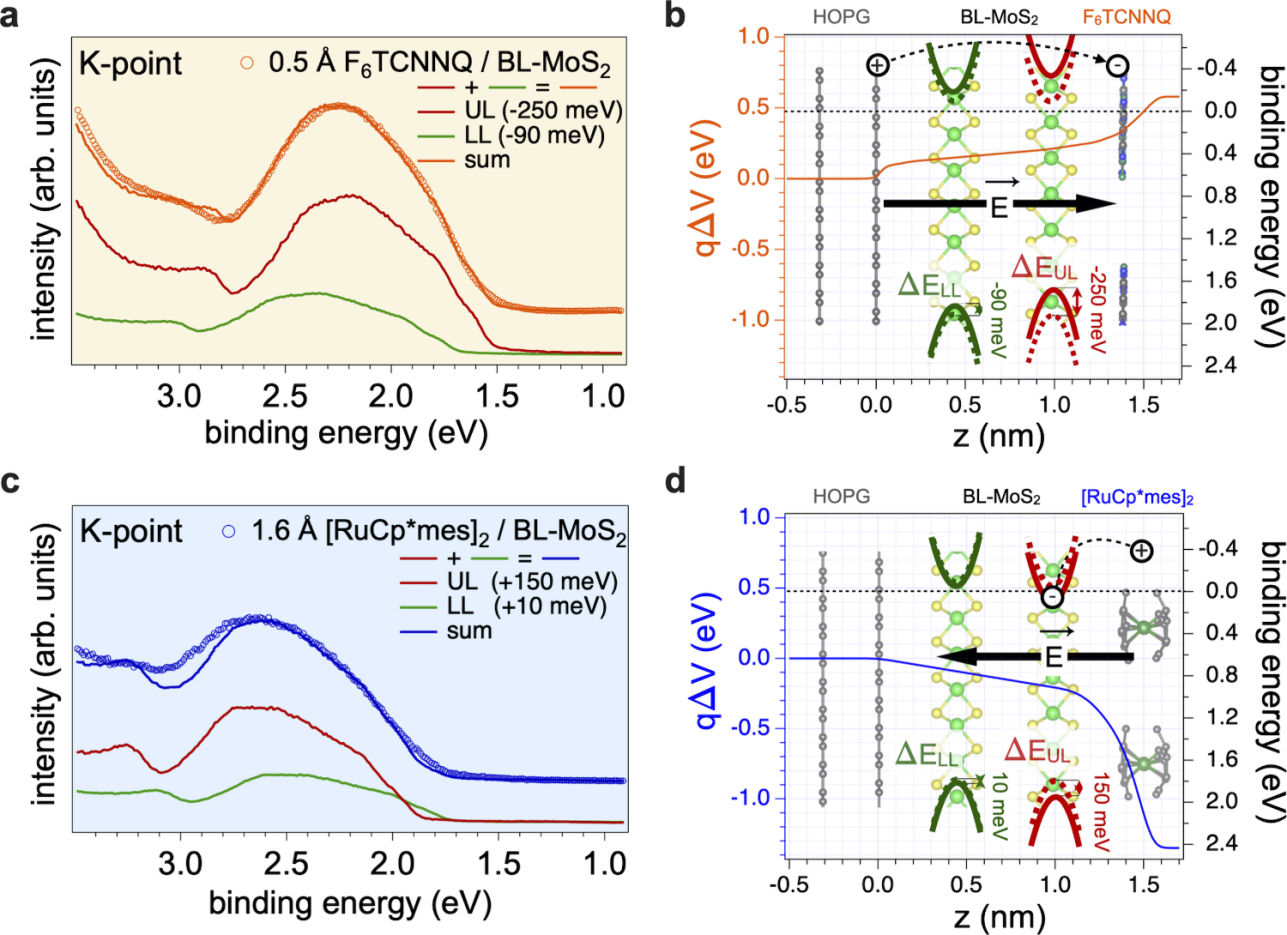
Energy-level realignment at the K-point and extracted electric-potential-energy landscape. Reconstruction of the (±10° integration momentum-integrated) EDC of the ARPES map at the K-point for (a) F_6_TCNNQ and (c) [RuCp*mes]_2_. Corresponding energy-level diagram for BL-MoS_2_ covered by (b) F_6_TCNNQ and (d) [RuCp*mes]_2_.

#### [RuCp*mes]_2_

The ARPES results for the deposition of 1.6 Å [RuCp*mes]_2_ are summarized in Figure 4e,f. Here, an overall shift to higher BE is noted, amounting to 145 meV for Γ_2_, concomitant with an increase in its width by 120% from ca. 150 to 330 meV. A partial filling of the BL-MoS_2_ conduction band (formed by Mo *d*_*z* ^2^_) caused by electron transfer from the donors is directly observed via the emergence of near-E_F_ DOS (with peak maximum at ca. 30 meV BE) around the K-point.^56^ This shows a strong n-type doping of the BL-MoS_2_ with an estimated carrier concentration of about 2 × 10^13^ cm^–2^.^56^ This is fully consistent with the XPS results, notably with the reduction of the Mo atoms concluded from the differential shift of the S 2p and Mo 3d core levels. The fact that the CBM is here observed at the K-point may be due to the initial stacking of the MoS_2_ layer, but given the random relative orientation of LL and UL, it is more likely an energy renormalization effect induced by the electric field.^34^ As for F_6_TCNNQ, the EDC of the valence spectrum at the K-point can be reconstructed by summing up the momentum integrated EDCs of the clean BL-MoS_2_, shifted by the same amount as the Mo 3d core levels, because the topmost valence band at K-point is dominated by the Mo *d*_*x* ^2^–*y* ^2^,*xy*_ contributions.^46^ The result of the EDC reconstruction shown in Figure 5c reveals again excellent agreement with the experiment. Figure 5d provides energy-level realignment occurring upon [RuCp*mes]_2_ deposition.

### Electric Fields at Higher Molecular Coverages

Figure 6 summarizes the BE shifts of the Mo 3d and S 2p core levels for the different F_6_TCNNQ and [RuCp*mes]_2_ coverages as a function of ΔWF together with the calculated electric fields. The XPS and ARPES analyses for the additional molecular deposition are provided in the SI (Figures S23–S30). For Figure 6, we calculated the magnitude of the electric field between the two MoS_2_ layers by considering the Stark splitting, i.e., by $E_{\mathrm{split}}=\frac{\Delta {\mathrm{BE}}_{\mathrm{UL}}-\Delta {\mathrm{BE}}_{\mathrm{LL}}}{e\Delta z_{\mathrm{UL}-\mathrm{LL}}}$ , with ΔBE_UL_ and ΔBE_LL_ being the binding energy shifts measured for LL and UL, *e* the electron charge and Δ*z*_UL‑LL_ = 6.6 Å the distance between the Mo atoms of LL and UL, respectively. *E*_split_ is the value usually reported in exciton studies and directly relates to the interlayer exciton energy shifts. At the maximum ΔWF included in this analysis of 0.75 eV, an ultrahigh electric field of ca. 0.34 V·nm^–1^ by F_6_TCNNQ-gating is comparable with literature values for the similar p-dopant molecule F_4_TCNQ.^26^

**Figure 6:**
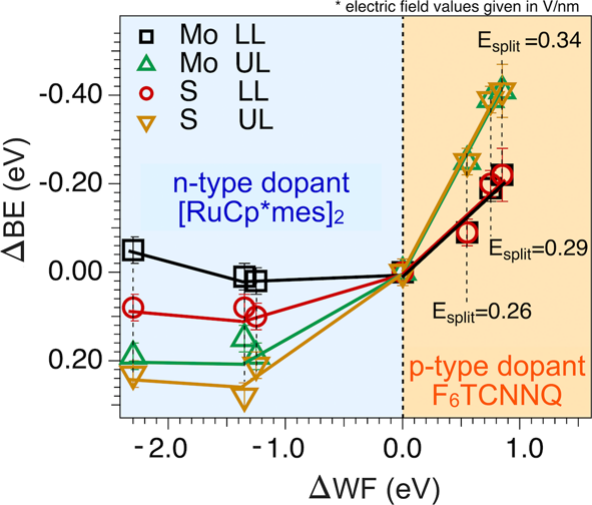
Stark shifts of each core-level component of BL-MoS_2_ (Mo and S for LL and UL) and corresponding ΔWF with emerged electrical fields. ΔBE of the Mo 3d and S 2p core levels determined for the different F_6_TCNNQ and [RuCp*mes]_2_ coverages as a function of ΔWF measured for different nominal molecular layer thicknesses. For F_6_TCNNQ, the electric field (*E*_split_) values (see the text) are also reported.

### Detailed Electric Potential Landscape, Charge Density Redistribution, and QCSE

So far, XPS and ARPES have helped clarify the change in electric potential affecting the two MoS_2_ layers upon molecular deposition. However, this approach implicitly assumed a constant potential *within* each MoS_2_ layer (see Figure S31). In the following, we propose that it is possible to go beyond this approximation by inspecting the details of the BE shifts and inhomogeneous broadening of the spectral function of the predominant valence feature Γ_2_ (Figure 4a). We reason that the observed change in the Γ_2_ line shape represents the broadening of the quasiparticle state in the potential gradient experienced by the atoms throughout the BL-MoS_2_,^54,55,57^ a feature that has been noticed for electrically gated TMDCs without attracting further attention.^37^ We now analyze the change in line shape of the Γ_2_ state toward tracking with unprecedented detail the potential profile along the surface-normal direction *z*. These insights into the potential landscape are then further used to determine the charge-density redistribution [Δρ­(*z*)] throughout the entire heterojunction, which is then employed as input to model the QCSE arising in these gated systems, a fundamental component for a comprehensive understanding of their optoelectronic properties.^15–17^

Figure 7a,b,d,e shows an overlay of the EDC of clean BL-MoS_2_ with the EDCs of BL-MoS_2_ covered with F_6_TCNNQ and [RuCp*mes]_2_, respectively. To minimize the possible impact of scattering effects of MoS_2_ photoelectrons by the molecular layer, only the momentum-integrated (±10° around the Γ) EDCs are presented. Note that the observations and reasoning described in the following for the momentum-integrated EDCs are actually independent of the integration region around Γ as the very same results are obtained for the momentum-resolved EDCs right at Γ (±0.1°) (Figure S35). In the displayed energy region, the EDCs exhibit two peaks at about 1.6 and 2.1 eV BE, the spectral signatures of the Γ_1_ and Γ_2_ QW states. Remarkably, modeling an infinite QW with a potential gradient allows us to accurately reproduce the energy shifts of these states (Figure S38). In our simulation, we found that, for [RuCp*mes]_2_, the large differential shift of ca. 75 meV between Γ_1_ and Γ_2_ is partly due to the large potential gradient and partly from a slight narrowing of the width of the well from 1.43 to 1.40 Å.

**Figure 7:**
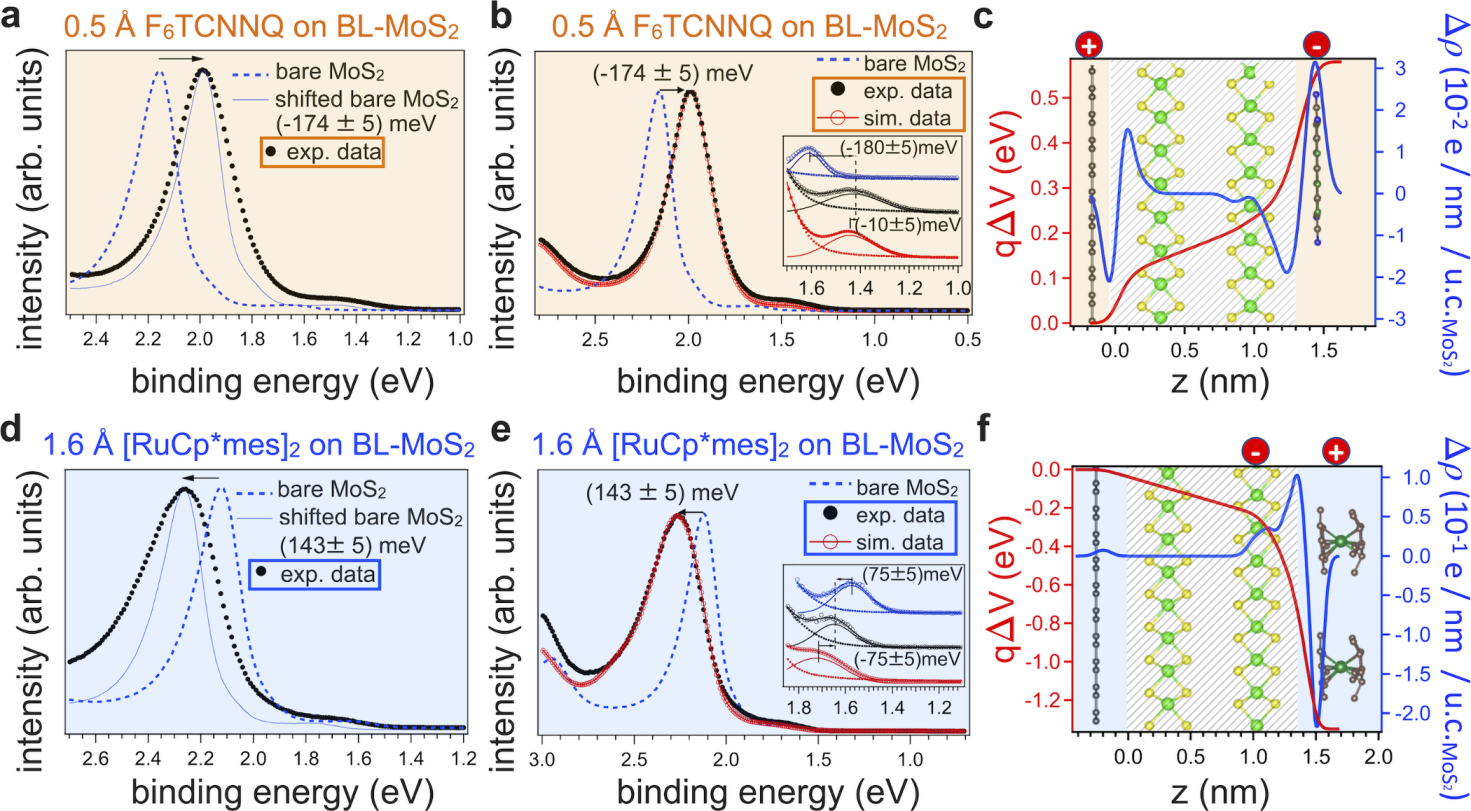
Valence-band EDC broadening and extracted potential gradients in BL-MoS_2_ upon molecular adsorption. Overlap of the ± 10° integrated EDCs at Γ before and after deposition of (a) F_6_TCNNQ and (d) [RuCp*mes]_2_ to highlight the change in line shape induced by molecular adsorption. As-measured clean and (b) F_6_TCNNQ and (e) [RuCp*mes]_2_ EDCs were obtained together with the simulated EDC obtained by convoluting the clean BL EDC with a function representing the potential gradient affecting the BL-MoS_2_ orbitals. Determined potential gradient and CD redistribution upon (c) F_6_TCNNQ and (f) [RuCp*mes]_2_ depositions for unit cell MoS_2_ (u.c._MoS2_). The curves in the dashed area region were determined from the shift and broadening of Γ_1,_ while outside this region, the potential was extrapolated to match the boundary conditions mentioned in the text and as detailed in SI.

In Figure 7a,d, an energetically shifted EDC for bare BL-MoS_2_ is also shown to help visualize the inhomogeneous line shape broadening, which is more pronounced toward the low BE side of the Γ_2_ peak for F_6_TCNNQ and toward the high BE side for [RuCp*mes]_2_, resulting in a strong peak asymmetry. This most likely results from the gradient in the electric potential in the out-of-plane direction. For a QW with a nonconstant potential *V­(z)*, the eigenstates are obtained by solving the corresponding Schrödinger equation:

$$\left\{\frac{\hbar^{2}}{2m^{*}}\frac{\partial^{2}}{\partial z^{2}}+q[V_{0}+V(z)]\right\}\Psi_{n}(E,z)=E_{n}\Psi_{n}(E,z)$$ 1

where *V*_0_ is the finite depth of the QW in the absence of field, *V*(*z*) is the varying potential resulting from the added field, and *q* is the elementary charge. In fact, exposing a QW to an out-of-plane electric field produces not only a Stark shift of the QW states but also their energy broadening. This arises from the fact that the formerly single (real) eigenvalues of the bound states acquire an imaginary part upon field exposure. In the case of a triangular well, this results in the emergence of Lorentzian resonances (so-called Breit-Wigner resonances), the width of which is determined by the imaginary part of the eigenvalue. Accordingly, the broadening of the resonances is intimately related to the shape and magnitude of *V*(*z*), and therefore the density of states *g*(*E*) of the QW can also be expressed as a function of *z*, such that

$$g(E,z)\approx g[E_{0}+qV(z),z]$$ 2

with *E*_0_ being the eigenenergy in the absence of a field.

Accordingly, neglecting photoemission matrix elements, the photoemission intensity as a function of energy *I*_PE_(*E*) is given by

$$I_{\mathrm{PE}}(E)=\int_{\mathrm{BL}}e^{-z/\lambda }\cdot I_{\mathrm{PE}}(E,z)dz\approx \int_{\mathrm{BL}}e^{-z/\lambda }\cdot g(E,z)dz\approx \int_{\mathrm{BL}}e^{-z/\lambda }\cdot g[E_{0}+qV(z),z]dz$$ 3

where λ is around 1.4 ML as determined above, and *g*(*E, z*) is the DOS stemming from each depth step d*z* across the BL-MoS_2_. When including a varying potential *V*(*z*), the energy of *g*(*E, z*) can also be written [*g*(*E*_0_ + *qV*)­(*z*), *z*], and *g* *(E)* is obtained by the *z*-integration of *g* *(E, z)* over the BL-MoS_2_ [indicated by BL in eq 3)]. *V­(z)* broadens and shifts *g* *(E),* yielding an accordingly shifted and broadened photoemission EDC.^58,59^

By comparison of the photoemission spectra before and after molecular adsorption, the potential gradient in the *z*-direction can then be traced back. The potential profile *V­(z)* is obtained by iteratively fitting the spectrum of the molecularly gated BL-MoS_2_ (*I*_PE,gated_^exp.^(*E*)) through a convolution product *I*_PE,gated_^simul.^(*E*) = *I*_PE,bare_^exp.^(*E*) ⊗ *f*(*z*), with $f(z)\propto {\left(\frac{dV(z)}{dz}\right)}^{-1}$ as detailed in the SI (Figures S33 and S34). The proportionality relation of *f*(*z*) with ${\left(\frac{dV(z)}{dz}\right)}^{-1}$ directly reflects the physics described by eq 3: Briefly, the smaller the potential gradient over a Δ*z* region, the more electronic states that accumulate at the same energy and the higher the photoemission intensity at this energy. Reciprocally, a large potential gradient results in an energy spread of the electronic states, and the photoemission intensity appears reduced but spread over a larger energy range, as detailed in the SI (Figures S33 and S34). The determined potential is not a function of the dielectric constant, though the charge redistribution leading to this potential is, as shown in the following. We stress that the same procedure can be successfully applied to simulate the S 2p core level spectra for the two heterojunctions by using the same *f*(*z*) as determined for the valence band region (Figure S36). The results of these procedures for F_6_TCNNQ and [RuCp*mes]_2_ are shown in Figure 7b,e, respectively, together with *V*(*z*) deduced from *f*(*z*) and depicted in the dashed region in Figure 7c,f. Outside the dashed regions, the potential gradient does not directly influence the MoS_2_ electronic states and was extrapolated in order to match the remaining ΔWF according to the boundary conditions given by the continuity of the potential at the bottom and top interfaces *V*|_in_ = *V*|_ext_ and its derivability $\frac{dV}{dz}{|}_{\mathrm{in}}=\frac{dV}{dz}{|}_{\mathrm{ext}}$ , where “in” corresponds to the MoS_2_ sides of the interfaces and “ext” corresponds to both the HOPG side and the molecular adsorbates side of the respective interfaces. The *V­(z)* profiles strongly differ for the two investigated cases. For F_6_TCNNQ, it is mostly linear through the BL-MoS_2_ due to the CT between HOPG and the F_6_TCNNQ, with a sharper drop on both sides of the MoS_2_ layer toward the HOPG and the molecular layer. This likely results from the polarization of the BL-MoS_2_ induced by the holes and electrons in the HOPG and F_6_TCNNQ, respectively. The linear potential drop, in which LL and UL undergo a potential change of ca. 140 ± 25 and 240 ± 50 mV, agrees well with the (90 ± 25) and (250 ± 25) meV BE shifts for LL and UL determined from the XPS fitting. In contrast, for [RuCp*mes]_2_, the potential slowly changes from HOPG up to the UL Mo atom, and it is then followed by a sharp drop close to the upper interface (which is responsible for the peak broadening on the high energy side of Γ_2_) consistent with the formation of a large interface dipole with the molecular layer.

The charge density redistribution is then derived by solving the one-dimensional Poisson equation:

$$\nabla^{2}V\left(z\right)=\frac{-\Delta \rho (z)}{\varepsilon_{0}\varepsilon \left(z\right)}$$ 4

with Δρ­(z), ε_0_ the vacuum permittivity, and ε­(*z*) the relative dielectric constant along the *z*-direction [here ε­(*z*) is chosen as a constant with a value of 6].^9,60–62^ Noteworthy, we here took an averaged, homogeneous value for ε­(*z*), close to that of intrinsic BL-MoS_2_ (ε_⊥_ ∼ 6.5) and HOPG (ε_⊥_∼ 6) and relevant for doped organic films (ε ∼ 3 to 15). Obviously, the biggest uncertainty is on the dielectric constant of the doped organic semiconductor, which would mostly affect the carrier concentration in the organic film but not necessarily the charge redistribution in the BL-MoS_2_. In the case of F_6_TCNNQ, the Δρ­(*z*) function clearly shows hole accumulation in the HOPG, partially screened by a small extent of electron accumulation in the lower part of the LL. Similarly, electron accumulation in the molecular region is partially screened by holes at the upper part of the UL, yielding the interface dipoles on each side of the BL-MoS_2_ that is seen in the corresponding potential function (Figure 7c). Concomitantly, BL-MoS_2_ remains approximately charge neutral. This results in an overall electron transfer from HOPG to the molecular layer of around 3 × 10^–2^ electrons per MoS_2_ unit cell (0.15 electrons per molecule for a 4 × 8 molecular arrangement with respect to the MoS_2_ lattice). The extent of CT derived here is roughly half of that calculated by density functional theory (DFT) for F_6_TCNNQ/ML-MoS_2_/HOPG, but the overall ΔWF is similar in both cases.^32^ ΔWF can be calculated according to the Helmholtz equation $\Delta \mathrm{WF}=\frac{qN\mu }{\varepsilon_{0}\varepsilon }$ , with *N* being the dipole density and μ the dipole moment perpendicular to the surface. As μ, which results from the CT between F_6_TCNNQ and HOPG, is expected to be the same in ML-MoS_2_ and BL-MoS_2_ (double distance but half the CT amount), our findings are in quantitative agreement with the work of Park et al.^32^ Remarkably, our experimentally derived Δρ­(*z*) shape displayed in Figure 7c accurately reflects the one calculated by DFT for ML-MoS_2_ in Park et al.^32^

In the case of [RuCp*mes]_2_, hole accumulation is found in the molecular layer together with a pronounced electron accumulation in MoS_2_, mostly in UL. The electron accumulation amounts to ca. 0.022 electrons and 0.001 electrons per MoS_2_ unit cell in the UL and LL, respectively, equivalent to carrier concentrations of ca. 2.4 × 10^13^ and 1.1 × 10^12^ cm^–2^. UL becomes highly negatively charged, while the HOPG substrate becomes only slightly charged. This demonstrates a strong electron transfer (ca. 0.65 electrons for the 4 × 8-based MoS_2_ unit cell molecular superstructure) from the molecules to UL, giving rise to a large interface dipole between these two components. In strong contrast to the F_6_TCNNQ case, BL-MoS_2_ is here directly involved in the CT process, a consequence of the MoS_2_ CBM being located initially just above the Fermi level and progressively going down in energy with the emerging electric field. As a result, the electron accumulation in UL largely screens the electric field between the molecular adsorbates and the HOPG substrate, leading to a relatively small potential drop through the LL and therefore a much lower carrier concentration in this layer. This charge redistribution pattern naturally correlates well with the negative charging of LL and UL as deduced from our XPS and ARPES measurements.

## Conclusions

We revealed the complex mechanisms of the energy level realignment of BL-MoS_2_ on HOPG by ultrahigh electric fields generated by molecular gating. The deposition of strong electron donor and acceptor molecules led to the emergence of electric fields up to ca. 0.35 V/nm, clearly overcoming the typical dielectric breakdown value of 0.15 V/nm^26,31^ and well in line with values deduced from differential reflectance measurements on similar structures. ARPES and angle-dependent XPS measurements enabled resolving the ground-state energy level realignment of each single component forming these heterostructures. A direct correlation of the EDCs around the K-point with the Stark-shifted Mo 3d and S 2p core levels was established through the reconstruction of the measured EDC. Analyzing the inhomogeneous broadening of the valence QW states around the Γ-point further allowed us to determine the potential landscape in the direction normal to the BL-MoS_2_. This was achieved via a semiclassical approach, including the convolution of the clean BL-MoS_2_ EDC with a function representing the potential gradient in the *z* direction and allowed simulating the EDC upon molecular gating. From the derived potential profile, the charge-density redistribution was then calculated and found to be in both qualitative and quantitative agreement with DFT calculations performed for a similar heterostructure but with ML-MoS_2_. Finally, the Mo *d*_*z* ^2^_ and S *p*_*z*_ derived QW states at the Γ-point were also analyzed in a fully quantum mechanical picture by solving the Schrödinger equation for a nonconstant infinite potential well, using the experimentally accessed potential landscape to reproduce the observed energy shift of these states. This suggests that the energy shift and line shape broadening of the QW states can be used for a precise determination of the electric potential profile at the atomic scale (see Figure S38). Another experimental means to access this inhomogeneous broadening of the QW states of the TMDC may be scanning tunneling spectroscopy (STS). However, the shift and broadening of these states probed with STS will eventually be influenced by the voltage applied between tip and sample, which will result in an additional electric field on the same order of magnitude as that resulting from the charge transfer due to molecular adsorption. Our work uncovers a full picture of the charge transfer and electronic phenomena involved in complex van der Waals heterojunctions. The disclosed understanding of the mechanisms leading to the ground-state electronic properties of TMDC-based heterostructures represents a key step in advancing the control of their excitonic properties. Specifically, to exploit Stark shifts for energy level tuning in bi- and multilayers of 2D materials, material selection to create the electric field must require going beyond simply considering ΔWF and IE/EA values of the components. As detailed in the main text, the charge on the molecular acceptor and especially on the molecular donor is screened by the TMDC itself. This process leads to specific changes of the potential landscape in the TMDC region. Therefore, the polarizability and dielectric screening are fundamental characteristics needed in order to extract the detailed potential landscapes in these heterostructures. Also, assuming that the CT-effects will be symmetric (i.e., for using donors or acceptors) can fail, as demonstrated in the present work, where the acceptor led to proper field drop across the BL and much less so with the donor. Furthermore, the enclosed 2D material cannot be readily taken as “bystander” to the CT but can be directly involved in the charge-density redistribution, as observed here for the molecular donor example. The gained knowledge is expected to have strong implications for the design and characteristics, e.g., of dual-gated transistors and the tuning of interlayer excitonic resonances in these devices, which relate to the (interlayer) bandgap of the biased BL-MoS_2_.^63^ Overall, our insights pave the way toward the implementation of 2D material-based next-generation field-effect electronic devices and the realization of exotic electronic phases, including Bose–Einstein condensates and excitonic insulating phases.

## Methods

### General

The ML-MoS_2_ was prepared by chemical vapor deposition (CVD)^64^ on sapphire as detailed below and then transferred onto a HOPG substrate (ZYA 0,4° 10 × 10 mm) by liquid transfer. A first ML-MoS_2_ was transferred and annealed (730 K at the sample position) in a preparation chamber (base pressure 2 × 10^–10^ mbar) in order to remove organic residuals from the transfer process and analyzed in situ in ultrahigh vacuum conditions (base pressure 1.10^–10^ mbar). A second ML-MoS_2_ was then deposited on top of the first one and annealed at 730 K. XPS and ARPES were used to ensure the cleanliness of the as-prepared sample. The organic molecules were evaporated in situ in a dedicated evaporation chamber (base pressure 5 × 10^–10^ mbar). The samples were not exposed to air between molecular deposition and photoelectron characterization.

### Characterization

The ARPES and XPS measurements were carried out with the PREVAC EA15 hemispherical energy analyzer. The monochromated He–Iα and monochromated Al–Kα radiations were provided by a helium discharge lamp equipped with a monochromator (UVS40A2, Prevac) and a monochromated X-ray source (RMC50, Prevac), respectively. The use of monochromated X-ray and UV sources with a typical transmission of about 10% for the characterization of these heterostructures allows us to avoid any molecular degradation during the photoemission measurements. In all ARPES and XPS spectra, the electron binding energies refer to the sample Fermi level. Secondary electron cutoff (SECO, for sample work function determination) was measured with a −10 V bias applied to the sample. The ARPES measurements were carried out with typical angular and energy experimental resolution of ∼0.1° and 20 meV, respectively. The experimental energy resolution in XPS was 0.3 eV.

Optical measurements were performed on an Olympus LEXT OLS 4100, with a lateral resolution of up to 0.12 μm. Raman measurements were performed on an XploRA-TM PLUS micro-Raman spectrometer for sample quality and coverage characterization with a green laser of 532 nm, detection slits of 100 μm, circular light polarization, and a grating of 2400 slits/mm.

### Materials

The ML-MoS_2_ (1.5 cm × 1.5 cm) was grown by CVD on preannealed (1270 K for 30 min) double-side polished (1.5 cm × 1.5 cm) sapphire (Siegert Wafer, AZ32033, *c*-plane (001), (430 ± 25) μm) using a two-zone furnace with a quartz tube of 50 mm in diameter. In the quartz cylindrical oven, solid sulfur was deposited at a distance of 17 cm from the cleaned sapphire substrate, on which a Mo-precursor was deposited through spin coating (Na_2_MoO_4_ in H_2_O at 0.015 mol/L at 300 rpm for 100 s). The quartz tube was then evacuated to ∼5 × 10^–1^ mbar and subsequently purged with argon gas (99.99% purity) until atmospheric condition is attained. The growth time was set to 5 min at the reaction temperature of 1100 K. The reaction temperature was reached through a first constant increase of the temperature over 20 min reaching 570 K, where the temperature was hold for 5 min and then increased until 1100 K over a period of 40 min. The argon carrier gas was used at 60 standard cubic centimeters per minute (sccm). After the reaction time, the oven was open as soon as the temperature within decreased to 1040 K.

The transfer of the synthesized ML-MoS_2_ from the sapphire to the HOPG was performed by liquid transfer using poly­(methyl methacrylate) (PMMA). PMMA was spin-coated at 1400 rpm for 100 s on as-grown ML-MoS_2_. The edges of the substrate were cut using a diamond cutter to expose the sapphire surface, allowing faster etching. Then, the substrate was floated up onto potassium hydroxide solution. The sapphire substrate was sunk into the solution after being etched off by the solution, and the PMMA film with MoS_2_ stayed on the surface. The film was transferred to deionized (DI) water 3–4 times to clean the residual solution before being fished out onto a fresh cleaved HOPG substrate (Optigraph GmbH, 10 × 10 mm) and dried at 120 °C for 20 min. After transfer of the first ML-MoS_2_ and before the transfer of the second layer, the sample was annealed at 670 K in UHV. The final BL-MoS_2_ on HOPG was then annealed at 730 K for 4 h.

[RuCp*mes]_2_ was synthesized as described in Un et al.^65^ and F_6_TCNNQ was purchased from Novaled. The molecules were then evaporated in situ from resistively heated crucibles.^66,67^ The molecular layer thickness was monitored with a quartz microbalance using a density of 1.3 g/cm^3^.

## Supplementary Material



## Data Availability

Data for this article are available at the Open-Access-Publikationsserver of Humboldt-Universität

## Notes

ARPES of ML- and BL-MoS_2_; optical microscopy, Raman spectroscopy and BL-MoS_2_ sample coverage estimation, XPS survey spectra; chemical structure of organic molecular charge donors; inelastic mean free path quantification; core level analysis with fitting procedure and two component model; band structure analysis for additional deposition thicknesses, hole effective mass estimation, convolution approach explanation, simulation of core level data by convolution approach, renormalization of the as-determined potential by the attenuation length from the inelastic mean free path; and quantum confined Stark effect and eigen energies at the Γ-point (PDF)

## References

[1] Manzeli S., Ovchinnikov D., Pasquier D., Yazyev O. V., Kis A. (2017). 2D Transition
Metal Dichalcogenides. Nat. Rev. Mater..

[2] Chhowalla M., Shin H. S., Eda G., Li L. J., Loh K. P., Zhang H. (2013). The Chemistry of Two-Dimensional
Layered Transition Metal Dichalcogenide
Nanosheets. Nat. Chem..

[3] Mak K. F., Shan J. (2016). Photonics and Optoelectronics
of 2D Semiconductor Transition Metal
Dichalcogenides. Nat. Photonics.

[4] Xu X., Yao W., Xiao D., Heinz T. F. (2014). Spin and Pseudospins in Layered Transition
Metal Dichalcogenides. Nat. Phys..

[5] Wang, H. ; Yu, L. ; Lee, Y. H. ; Fang, W. ; Hsu, A. ; Herring, P. ; Chin, M. ; Dubey, M. ; Li, L. J. ; Kong, J. ; Palacios, T. Large-Scale 2D Electronics Based on Single-Layer MoS2 Grown by Chemical Vapor Deposition. In Technical Digest - International Electron Devices Meeting IEDM, 2012.

[6] Dodda A., Jayachandran D., Pannone A., Trainor N., Stepanoff S. P., Steves M. A., Radhakrishnan S. S., Bachu S., Ordonez C. W., Shallenberger J. R., Redwing J. M., Knappenberger K. L., Wolfe D. E., Das S. (2022). Active Pixel
Sensor Matrix Based
on Monolayer MoS2 Phototransistor Array. Nat.
Mater..

[7] Xiao D., Liu G. B., Feng W., Xu X., Yao W. (2012). Coupled Spin
and Valley Physics in Monolayers of MoS 2 and Other Group-VI Dichalcogenides. Phys. Rev. Lett..

[8] Mak K. F., Lee C., Hone J., Shan J., Heinz T. F. (2010). Atomically Thin
MoS 2: A New Direct-Gap Semiconductor. Phys.
Rev. Lett..

[9] Cheiwchanchamnangij T., Lambrecht W. R. L. (2012). Quasiparticle Band Structure Calculation of Monolayer,
Bilayer, and Bulk MoS 2. Phys. Rev. B - Condens.
Matter Mater. Phys..

[10] Lee J., Mak K. F., Shan J. (2016). Electrical Control of the Valley
Hall Effect in Bilayer MoS2 Transistors. Nat.
Nanotechnol..

[11] Du L., Liao M., Liu G. B., Wang Q., Yang R., Shi D., Yao Y., Zhang G. (2019). Strongly Distinct Electrical Response
between Circular and Valley Polarization in Bilayer Transition Metal
Dichalcogenides. Phys. Rev. B.

[12] Park S., Mutz N., Schultz T., Blumstengel S., Han A., Aljarb A., Li L. J., List-Kratochvil E. J. W., Amsalem P., Koch N. (2018). Direct Determination of Monolayer
MoS2 and WSe2 Exciton Binding Energies on Insulating and Metallic
Substrates. 2D Mater..

[13] Chernikov A., Berkelbach T. C., Hill H. M., Rigosi A., Li Y., Aslan O. B., Reichman D. R., Hybertsen M. S., Heinz T. F. (2014). Exciton Binding
Energy and Nonhydrogenic Rydberg Series
in Monolayer WS2. Phys. Rev. Lett..

[14] Molas M. R., Slobodeniuk A. O., Nogajewski K., Bartos M., Bala, Babiński A., Watanabe K., Taniguchi T., Faugeras C., Potemski M. (2019). Energy Spectrum
of Two-Dimensional Excitons in a Nonuniform Dielectric Medium. Phys. Rev. Lett..

[15] Roch J. G., Leisgang N., Froehlicher G., Makk P., Watanabe K., Taniguchi T., Schönenberger C., Warburton R. J. (2018). Quantum-Confined
Stark Effect in a MoS2Monolayer van Der Waals Heterostructure. Nano Lett..

[16] Chakraborty C., Goodfellow K. M., Dhara S., Yoshimura A., Meunier V., Vamivakas A. N. (2017). Quantum-Confined Stark Effect of
Individual Defects in a van Der Waals Heterostructure. Nano Lett..

[17] Klein J., Wierzbowski J., Regler A., Becker J., Heimbach F., Müller K., Kaniber M., Finley J. J. (2016). Stark Effect
Spectroscopy
of Mono- and Few-Layer MoS2. Nano Lett..

[18] Wang Z., Chiu Y. H., Honz K., Mak K. F., Shan J. (2018). Electrical
Tuning of Interlayer Exciton Gases in WSe2 Bilayers. Nano Lett..

[19] Miller B., Steinhoff A., Pano B., Klein J., Jahnke F., Holleitner A., Wurstbauer U. (2017). Long-Lived
Direct and Indirect Interlayer
Excitons in van Der Waals Heterostructures. Nano Lett..

[20] Rivera P., Schaibley J. R., Jones A. M., Ross J. S., Wu S., Aivazian G., Klement P., Seyler K., Clark G., Ghimire N. J., Yan J., Mandrus D. G., Yao W., Xu X. (2015). Observation of Long-Lived Interlayer Excitons in Monolayer MoSe2–WSe2
Heterostructures. Nat. Commun..

[21] Jauregui L. A., Joe A. Y., Pistunova K., Wild D. S., High A. A., Zhou Y., Scuri G., de Greve K., Sushko A., Yu C. H., Taniguchi T., Watanabe K., Needleman D. J., Lukin M. D., Park H., Kim P. (2019). Electrical Control
of Interlayer Exciton Dynamics in Atomically Thin Heterostructures. Science.

[22] Unuchek D., Ciarrocchi A., Avsar A., Watanabe K., Taniguchi T., Kis A. (2018). Room-Temperature Electrical Control of Exciton Flux in a van Der
Waals Heterostructure. Nat..

[23] Xu Y., Kang K., Watanabe K., Taniguchi T., Mak K. F., Shan J. (2022). Tunable Bilayer Hubbard
Model Physics
in Twisted WSe2. Nat. Nanotechnol..

[24] Montblanch A. R. P., Kara D. M., Paradisanos I., Purser C. M., Feuer M. S. G., Alexeev E. M., Stefan L., Qin Y., Blei M., Wang G., Cadore A. R., Latawiec P., Lončar M., Tongay S., Ferrari A. C., Atatüre M. (2021). Confinement
of Long-Lived Interlayer Excitons in WS2/WSe2 Heterostructures. Commun. Phys..

[25] Peimyoo N., Deilmann T., Withers F., Escolar J., Nutting D., Taniguchi T., Watanabe K., Taghizadeh A., Craciun M. F., Thygesen K. S., Russo S. (2021). Electrical Tuning of
Optically Active Interlayer Excitons in Bilayer MoS2. Nat. Nanotechnol..

[26] Bolotin, K. ; Kovalchuk, S. ; Greben, K. ; Kumar, A. ; Pessel, S. ; Soyka, J. ; Cao, Q. ; Watanabe, K. ; Taniguchi, T. ; Christiansen, D. ; Selig, M. ; Knorr, A. ; Siegfried, E. New Interlayer Excitons in 2D Bilayers Revealed under Strong Electric Field. Res. Square (Preprint) 2023. 10.21203/rs.3.rs-3453831/v1.

[27] Wang Z., rhodes D. A., Watanabe K., Taniguchi T., Hone J. C., Shan J., Fai Mak K. (2019). Evidence of
High-Temperature
Exciton Condensation in Two-Dimensional Atomic Double Layers. Nature.

[28] Xie M., Pan H., Wu F., Das Sarma S. (2023). Nematic Excitonic Insulator in Transition
Metal Dichalcogenide Moiré Heterobilayers. Phys. Rev. Lett..

[29] Chen D., Lian Z., Huang X., Su Y., Rashetnia M., Ma L., Yan L., Blei M., Xiang L., Taniguchi T., Watanabe K., Tongay S., Smirnov D., Wang Z., Zhang C., Cui Y. T., Shi S. F. (2022). Excitonic Insulator
in a Heterojunction Moiré Superlattice. Nat. Phys..

[30] Zhang Z., Regan E. C., Wang D., Zhao W., Wang S., Sayyad M., Yumigeta K., Watanabe K., Taniguchi T., Tongay S., Crommie M., Zettl A., Zaletel M. P., Wang F. (2022). Correlated Interlayer Exciton Insulator in Heterostructures of Monolayer
WSe2 and Moiré WS2/WSe2. Nat. Phys..

[31] Hattori Y., Taniguchi T., Watanabe K., Nagashio K. (2016). Anisotropic Dielectric
Breakdown Strength of Single Crystal Hexagonal Boron Nitride. ACS Appl. Mater. Interfaces.

[32] Park S., Wang H., Schultz T., Shin D., Ovsyannikov R., Zacharias M., Maksimov D., Meissner M., Hasegawa Y., Yamaguchi T., Kera S., Aljarb A., Hakami M., Li L.-J., Tung V., Amsalem P., Rossi M., Koch N. (2021). Temperature-Dependent
Electronic Ground-State Charge Transfer in
van Der Waals Heterostructures. Adv. Mater..

[33] Park S., Schultz T., Xu X., Wegner B., Aljarb A., Han A., Li L. J., Tung V. C., Amsalem P., Koch N. (2019). Demonstration
of the Key Substrate-Dependent Charge Transfer Mechanisms between
Monolayer MoS2 and Molecular Dopants. Commun.
Phys..

[34] Kang M., Kim B., Ryu S. H., Jung S. W., Kim J., Moreschini L., Jozwiak C., Rotenberg E., Bostwick A., Kim K. S. (2017). Universal
Mechanism of Band-Gap Engineering in Transition-Metal Dichalcogenides. Nano Lett..

[35] Jones A. J. H., Muzzio R., Pakdel S., Biswas D., Curcio D., Lanata N., Hofmann P., McCreary K. M., Jonker B. T., Watanabe K., Taniguchi T., Singh S., Koch R. J., Jozwiak C., Rotenberg E., Bostwick A., Miwa J. A., Katoch J., Ulstrup S. (2022). Visualizing
Band Structure Hybridization
and Superlattice Effects in Twisted MoS2/WS2 Heterobilayers. 2D Mater..

[36] Parashar B., Rathmann L., Kim H. J., Cojocariu I., Bostwick A., Jozwiak C., Rotenberg E., Avila J., Dudin P., Feyer V., Stampfer C., Beschoten B., Bihlmayer G., Schneider C. M., Plucinski L. (2023). Photoemission Study of Twisted Monolayers and Bilayers
of WSe2 on Graphite Substrates. Phys. Rev. Mater..

[37] Hofmann P. (2021). Accessing
the Spectral Function of in Operando Devices by Angle-Resolved Photoemission
Spectroscopy. AVS Quantum Sci..

[38] Trainer D. J., Putilov A. V., Di Giorgio C., Saari T., Wang B., Wolak M., Chandrasena R. U., Lane C., Chang T. R., Jeng H. T., Lin H., Kronast F., Gray A. X., Xi X. X., Nieminen J., Bansil A., Iavarone M. (2017). Inter-Layer
Coupling Induced Valence Band Edge Shift in Mono- to Few-Layer MoS2. Sci. Rep..

[39] Park S., Schultz T., Han A., Aljarb A., Xu X., Beyer P., Opitz A., Ovsyannikov R., Li L. J., Meissner M., Yamaguchi T., Kera S., Amsalem P., Koch N. (2019). Electronic Band Dispersion
Determination in Azimuthally Disordered Transition-Metal Dichalcogenide
Monolayers. Commun. Phys..

[40] Zhang F., Kahn A., Zhang F., Kahn A. (2018). Investigation of the
High Electron Affinity Molecular Dopant F6-TCNNQ for Hole-Transport
Materials. Adv. Funct. Mater..

[41] Smith H. L., Dull J. T., Longhi E., Barlow S., Rand B. P., Marder S. R., Kahn A. (2020). N-Doping of a Low-Electron-Affinity
Polymer Used as an Electron-Transport Layer in Organic Light-Emitting
Diodes. Adv. Funct. Mater..

[42] Lin X., Wegner B., Lee K. M., Fusella M. A., Zhang F., Moudgil K., Rand B. P., Barlow S., Marder S. R., Koch N., Kahn A. (2017). Beating the Thermodynamic Limit with
Photo-Activation of n-Doping in Organic Semiconductors. Nat. Mater..

[43] Song Z., Schultz T., Ding Z., Lei B., Han C., Amsalem P., Lin T., Chi D., Wong S. L., Zheng Y. J., Li M. Y., Li L. J., Chen W., Koch N., Huang Y. L., Wee A. T. S. (2017). Electronic Properties
of a 1D Intrinsic/p-Doped Heterojunction in a 2D Transition Metal
Dichalcogenide Semiconductor. ACS Nano.

[44] Amsalem P., Heimel G., Koch N. (2018). Experimental
Investigation on Charge
Transfer Between Organic Adsorbates and Solid Surfaces. Encycl. Interfacial Chem. Surf. Sci. Electrochem..

[45] Amsalem P., Niederhausen J., Wilke A., Heimel G., Schlesinger R., Winkler S., Vollmer A., Rabe J. P., Koch N. (2013). Role of Charge
Transfer, Dipole-Dipole Interactions, and Electrostatics in Fermi-Level
Pinning at a Molecular Heterojunction on a Metal Surface. Phys. Rev. B.

[46] Cheng, Y. ; Schwingenschlögl, U. MoS2: A First-Principles Perspective. In MoS2. Lecture Notes in Nanoscale Science and Technology; Wang, Z. , Eds.; Springer: Cham, 2014; pp. 103–128.

[47] Zibouche N., Schlipf M., Giustino F. (2021). GW Band Structure of Monolayer MoS2
Using the SternheimerGW Method and Effect of Dielectric Environment. Phys. Rev. B.

[48] Koller G., Berkebile S., Oehzelt M., Puschnig P., Ambrosch-Draxl C., Netzer F. P., Ramsey M. G. (2007). Intra- and Intermodular
Band Dispersion
in an Organic Crystal. Science.

[49] Davies, P. C. W. ; Betts, D. S. Wave mechanics 1. In: Quantum Mechanics; Dobbs, E. R. ; Palmer, S. B. , Eds.; CRC Press: Boca Raton, 2002; pp. 15–26.

[50] Gatsios C., Dreher M., Amsalem P., Opitz A., Jouclas R., Geerts Y., Witte G., Koch N. (2024). Two Isomeric
Thienoacenes
in Thin Films: Unveiling the Influence of Molecular Structure and
Intermolecular Packing on Electronic Properties. J. Phys. Chem. C.

[51] Giovanelli L., Bocquet F. C., Amsalem P., Lee H. L., Abel M., Clair S., Koudia M., Faury T., Petaccia L., Topwal D., Salomon E., Angot T., Cafolla A. A., Koch N., Porte L., Goldoni A., Themlin J. M. (2013). Interpretation
of Valence Band Photoemission Spectra at Organic-Metal Interfaces. Phys. Rev. B - Condens. Matter Mater. Phys..

[52] Bocquet F. C., Giovanelli L., Amsalem P., Petaccia L., Topwal D., Gorovikov S., Abel M., Koch N., Porte L., Goldoni A., Themlin J. M. (2011). Final-State Diffraction Effects in
Angle-Resolved Photoemission at an Organic-Metal Interface. Phys. Rev. B - Condens. Matter Mater. Phys..

[53] Giovanelli L., Amsalem P., Angot T., Petaccia L., Gorovikov S., Porte L., Goldoni A., Themlin J. M. (2010). Valence Band Photoemission
from the Zn-Phthalocyanine/Ag(110) Interface: Charge Transfer and
Scattering of Substrate Photoelectrons. Phys.
Rev. B - Condens. Matter Mater. Phys..

[54] Cimino R., Giarante A., Horn K., Pedio M. (1995). Line Broadening in
Semiconductor Core Level Photoemission Induced by Barrier Height Inhomogeneity. Surf. Sci..

[55] Lev L. L., Maiboroda I. O., Grichuk E. S., Chumakov N. K., Schröter N. B. M., Husanu M. A., Schmitt T., Aeppli G., Zanaveskin M. L., Valeyev V. G., Strocov V. N. (2022). Impact
of Band-Bending on the k-Resolved
Electronic Structure of Si-Doped GaN. Phys.
Rev. Res..

[56] Caruso F., Amsalem P., Ma J., Aljarb A., Schultz T., Zacharias M., Tung V., Koch N., Draxl C. (2021). Two-Dimensional
Plasmonic Polarons in n -Doped Monolayer MoS2. Phys. Rev. B.

[57] Rissner F., Egger D. A., Natan A., Körzdörfer T., Kümmel S., Kronik L., Zojer E. (2011). Collectively Induced
Quantum-Confined Stark Effect in Monolayers of Molecules Consisting
of Polar Repeating Units. J. Am. Chem. Soc..

[58] Lazarenkova O. L., Pikhtin A. N. (1998). Energy Spectrum
of a Nonideal Quantum Well in an Electric
Field. Semiconductors.

[59] Trzeciakowski W., Gurioli M. (1991). Electric-Field Effects
in Semiconductor Quantum Wells. Phys. Rev. B.

[60] Jellison G. E., Hunn J. D., Lee H. N. (2007). Measurement
of Optical Functions
of Highly Oriented Pyrolytic Graphite in the Visible. Phys. Rev. B - Condens. Matter Mater. Phys..

[61] Warren R., Blom P. W. M., Koch N. (2023). Molecular
p -Doping Induced Dielectric
Constant Increase of Polythiophene Films Determined by Impedance Spectroscopy. Appl. Phys. Lett..

[62] Yang X., Liu J., Koster L. J. A. (2024). The
Exceptionally High Dielectric Constant of Doped
Organic Semiconductors. Adv. Electron. Mater..

[63] Chu T., Ilatikhameneh H., Klimeck G., Rahman R., Chen Z. (2015). Electrically
Tunable Bandgaps in Bilayer MoS2. Nano Lett..

[64] Sharma R., Nameirakpam H., Muradas Belinchón D., Sharma P., Noumbe U., Belotcerkovtceva D., Berggren E., Vretenár V., Vanco L., Matko M., Biroju R. K., Satapathi S., Edvinsson T., Lindblad A., Kamalakar M. V. (2024). Large-Scale
Direct Growth of Monolayer MoS_2_ on Patterned Graphene for
van der Waals Ultrafast Photoactive Circuits. ACS Appl. Mater. Interfaces.

[65] Un H. I., Gregory S. A., Mohapatra S. K., Xiong M., Longhi E., Lu Y., Rigin S., Jhulki S., Yang C. Y., Timofeeva T. V., Wang J. Y., Yee S. K., Barlow S., Marder S. R., Pei J. (2019). Understanding the Effects of Molecular Dopant on N-Type Organic Thermoelectric
Properties. Adv. Energy Mater..

[66] Wang R., Schultz T., Papadogianni A., Longhi E., Gatsios C., Zu F., Zhai T., Barlow S., Marder S. R., Bierwagen O., Amsalem P., Koch N. (2023). Tuning the Surface Electron Accumulation
Layer of In2O3 by Adsorption of Molecular Electron Donors and Acceptors. Small.

[67] Untilova V., Zeng H., Durand P., Herrmann L., Leclerc N., Brinkmann M. (2021). Intercalation
and Ordering of F6TCNNQ and F4TCNQ Dopants
in Regioregular Poly­(3-Hexylthiophene) Crystals: Impact on Anisotropic
Thermoelectric Properties of Oriented Thin Films. Macromolecules.

