# Supplementary material for: Atomic-Scale Electric Potential Landscape across Molecularly Gated Bilayer MoS2 Resolved by Photoemission
Source: ACS Nano. 2025 Sep 8;19(36):32693–704. doi: 10.1021/acsnano.5c10363 (PMC12445001; doi:10.1021/acsnano.5c10363)
Supplement: Supplementary file 1 [file nn5c10363_si_001.pdf]

Supporting Information for:

Atomic-scale electric potential landscape across molecularly gated bilayer MoS_2 resolved by photoemission

Laura Scholz[1], Patrick Amsalem*[1], Lennart Frohloff[1], Rongbin Wang[1], Emily Albert[1,2], Kan Tang[3], Stephen Barlow[3], Seth R. Marder[3,4], and Norbert Koch*[1,2]

[1]Insitut für Physik & Center for the Science of Materials Berlin, Humboldt-Universität zu Berlin, Berlin, 12489 Berlin, Germany

[2]Helmholtz-Zentrum Berlin für Materialien und Energie GmbH, Berlin, 12489 Berlin, Germany

[3]Renewable and Sustainable Energy Institute (RASEI), University of Colorado Boulder, Boulder, 80309 Colorado, United States

[4]Department of Chemical and Biological Engineering and Department of Chemistry, University of Colorado Boulder, Boulder, 80309 Colorado, United States

* amsalem@physik.hu-berlin.de, norbert.koch@physik.hu-berlin.de

1. *ML and BL MoS_2 on HOPG*

Before characterization of the energetic levels of the bilayer (BL-) MoS_2 in the 2H phase under the influence of molecular layers, the transferred monolayer (ML) on highly oriented pyrolytic graphite (HOPG) and later also the BL-MoS_2 on HOPG were characterized to determine the quality and energy level alignment of the sample.

a. *Energy level alignment*

Bulk MoS_2 is an indirect bandgap semiconductor (SC), the direct excitonic transition sits at around 1.7 eV at the K-point. Reducing the materials thickness, and therefore the layer number, increases the indirect bandgap, while the features at the K-point remain nearly unchanged. In the ML limit MoS_2 becomes a direct bandgap SC.[1]

Fig. S1 shows the experimentally obtained band dispersion of ML and BL- MoS_2 on HOPG at the Γ- and K-point of the in-house fabricated samples acquired through angle resolved photoemission spectroscopy (ARPES) measurements.

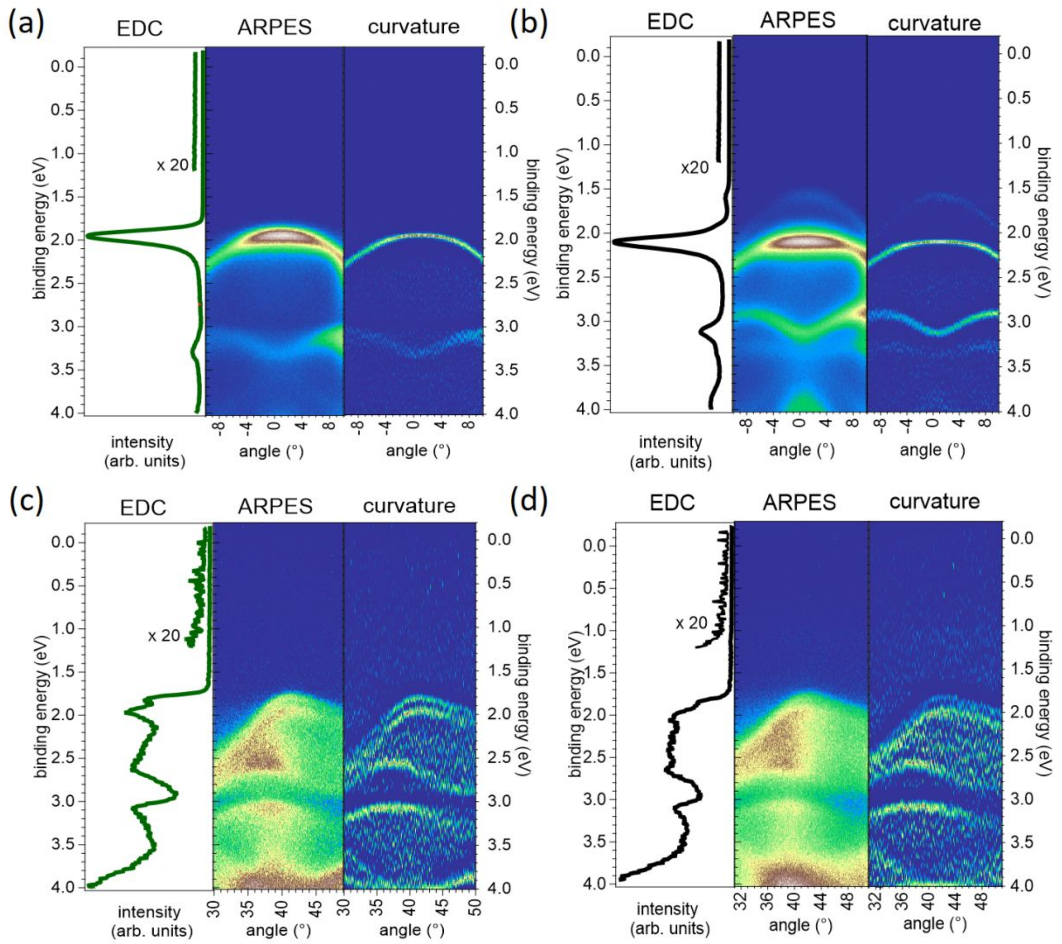

Fig. S1: Experimental EDC, ARPES and ARPES-curvature data of ML-MoS_2 at the reciprocal lattice point Γ (a) and K (c), and BL-MoS_2 at the reciprocal lattice points Γ (b) and K (d). The plotted binding energy is given relative to the Fermi level (E_F). The additional data points in each EDC graph between 0.2 eV and 1.2 eV represent an intensity increase of 2000% of the EDC-data found in that binding energy range.

As expected for TMDCs, a shift from direct to indirect semiconductor from the ML limit to the BL is observed. Through image manipulation the curvature of the ARPES data is shown on the right hand side were the spin orbit splitting found at the K-point is nicely recognizable. The K-point shift to lower binding energies for the BL, finding K at 1.70 eV and 1.89 eV, in comparison to the ML with K at 1.84 eV and 1.97 eV. The band structure at K-point is dominated by the in plane Mo d-orbitals, which are unaffected by the quantum confinement change caused by the additional layer when passing from a ML to a BL configuration[2]. At the Γ-point the ML shows one clear band sitting at 1.96 eV, while the BL shows, a double feature, which sit at 1.61 eV and 2.10 eV. This change is due to quantum confinement: in fact, the band around the Γ-point are characterized by the hybridization between the S p_z-orbitals and the out of plane Mo d_{z^2}-orbitals which emerges by changing the number of layers[3].

b. *Sample coverage determination: optical microscopy and Raman measurements*

As described in the experimental part of the paper the single ML-MoS_2 of size 0.9 cm x 0.9 cm were transferred in liquid solution onto a 1 cm^2 HOPG substrate. The smaller area was needed to ensure no overlap at the edge of the substrate. The defects caused by the screws needed to stabilize the sample in the measuring setup, as well as defects caused in the growing process needed to be evaluated and quantified. The sample was therefore analyzed under optical microscopy and underwent Raman measurements in order to determine the ML, BL and graphite ratios within the sample. An optical microscope image is shown in Fig. S2. The total BL coverage in comparison to the total MoS_2 coverage on the sample found through the analysis with the Gwyddion software equals 79 %, while the ML to BL ratio is around 26%, the total MoS_2 coverage on the substrate is 83%. All values are given with an accuracy of 3% of the total coverage. The center region (inner 0.5 cm x 0.5 cm region), therefore the region in which later UPS, XPS, and ARPES measurements were performed, showed a BL coverage of above 95%. The Raman measurement results are shown in Table S1. In order to characterize the sample around 5 different spots in each of the four quadrants of the sample, as well as 8 points in the centre region of the sample were taken, the results are shown in Fig. S4-S13 and summarized in Fig. S3. As shown, the results are consistent with the literature values given in Pierucci et al.[4] Fig. S3.f shows the correlation between the frequency of the in plane E_{2g} and out-of plane A_{1g} phonon mode for different spots taken at the four edges and in the centre region of the CVD grown and liquid transferred BL-MoS_2 on HOPG. As can be clearly observed, the correlations are recognized to follow, up to small dispersions within the margin of error, direct proportionality lines of the two phonon modes ($\omega(A^1_{1g})-\omega(E^1_{2g})$=const.). Three main offset values along which the Raman peaks disperse are found: $\omega(A^1_{1g})-\omega(E^1_{2g})=21\ cm^{-1}$ and 23 cm^{-1} and 25 cm^{-1}, related to the ML, BL and TL MoS_2 as stated in Pierucci et al.(2016)[4]. As both modes are equally subject to strain[5–9], this indicates that a Gaussian strain distribution is found in the sample. On the other hand, as only the A_{1g} peak is affected by charged doping of the material[6], the negligible dispersion along the normal direction to the strain dispersion indicates a homogenous doping of the BL-MoS_2 over the whole sample.

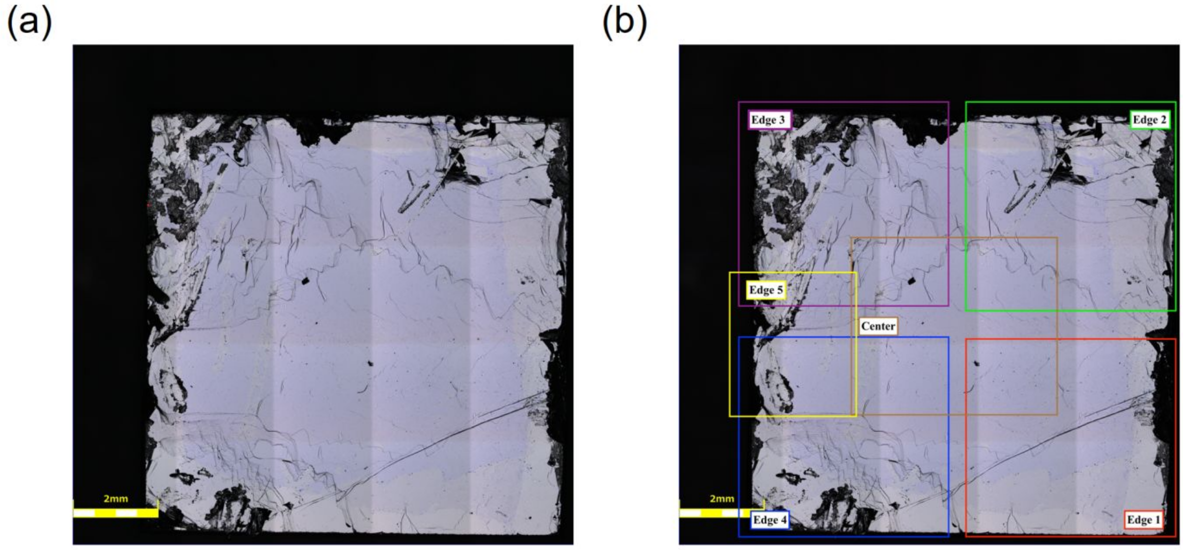

Fig. S2: Optical microscopy map of BL-MoS_2 sample for a magnification factor of 5. Both subfigures show the same image, but subFig. (b) portrays additionally the defined regions, used later for the Raman measurements. The observable lines that

composed the 5x5 structure over the map are processing artefacts of the microscope software, which occur upon superposition of the single snapshots. The contrast regions can be nicely depicted in the Edge 1 region, where bare substrate, on the sample edges (grey colour), ML-MoS_2 in a trigonal shape (light blue) and B BL-MoS_2 (dark blue) are clearly observable.

		E^1_{2g}-peak (cm^{-1})	A_{1g}-peak (cm^{-1})	difference (cm^{-1})
ML	literature value			20.0
	experiment	382.7 ± 0.4	403.2	20.5 ± 0.2
BL	literature value			22.0
	experiment	382.2 ± 0.4	404.4	22.2 ± 0.3
multilayer	literature value			>25
	experiment	<381.7	>405.6	>23.9

Table S1: Summary of literature (Pierucci et al. (2016)[4]) and experimentally found values of Raman characterisation of the clean BL-MoS_2 sample.

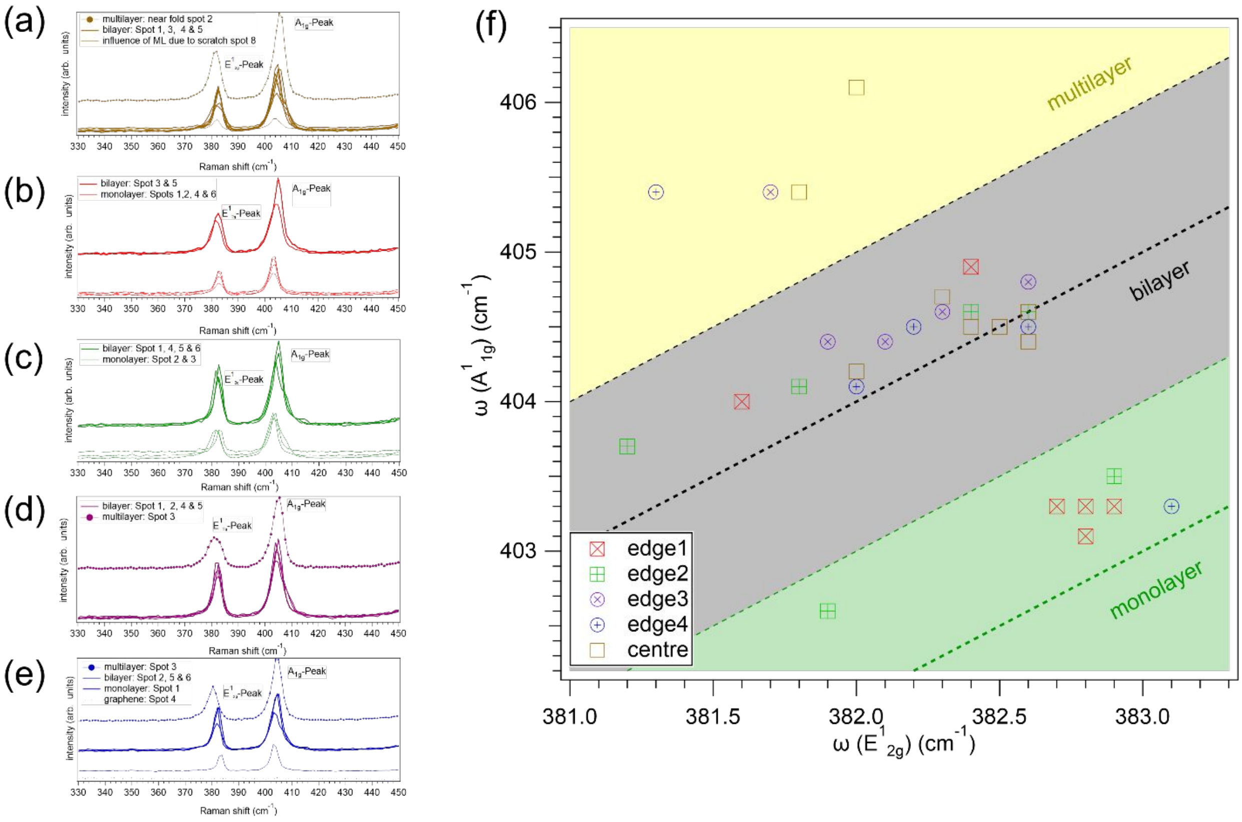

Fig. S3: Raman spectra found for predicted layer numbers at the centre (a), edge 1(b), 2(c), 3(d) and 4(e). (f) shows the distribution of the measured frequency of the A_{1g} and E_{2g} phonon mode for different spots of the four edges and centre region of the CVD grown and liquidly transferred BL-MoS_2/HOPG sample. ML., BL and TL/MuL region are shown in green grey and yellow areas

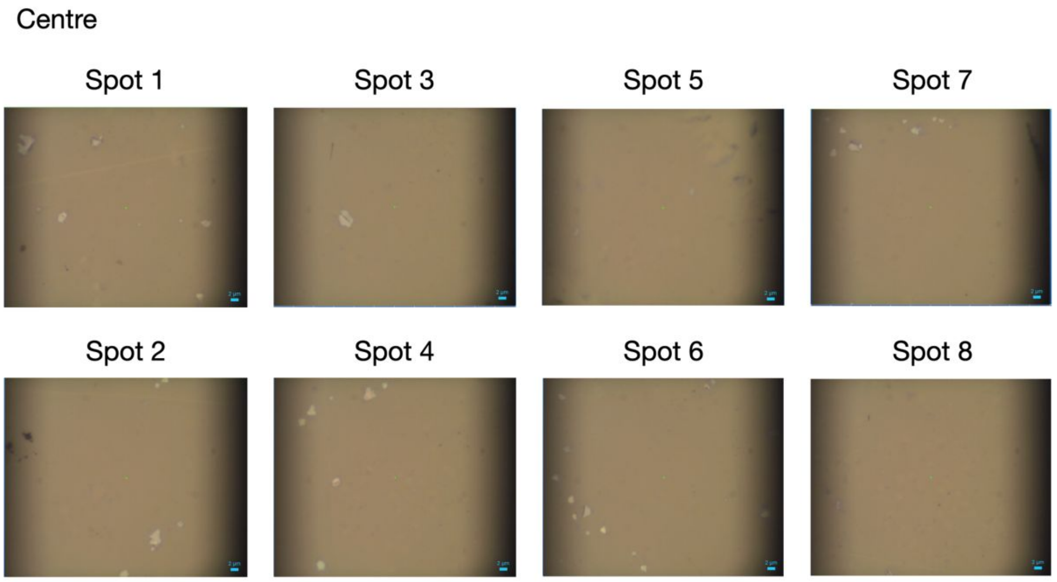

Fig. S4: Optical microscopy image of different spots in the center region.

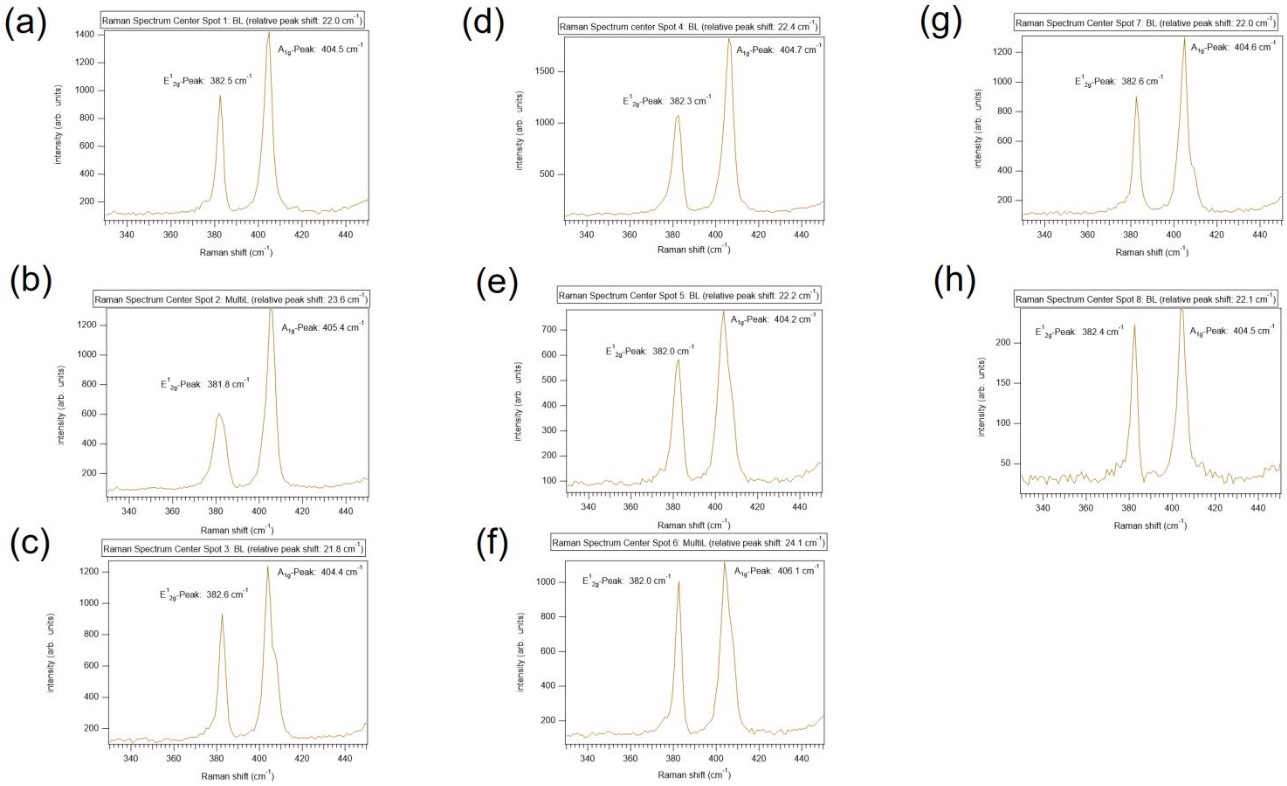

Fig. S5: Raman spectra center region.

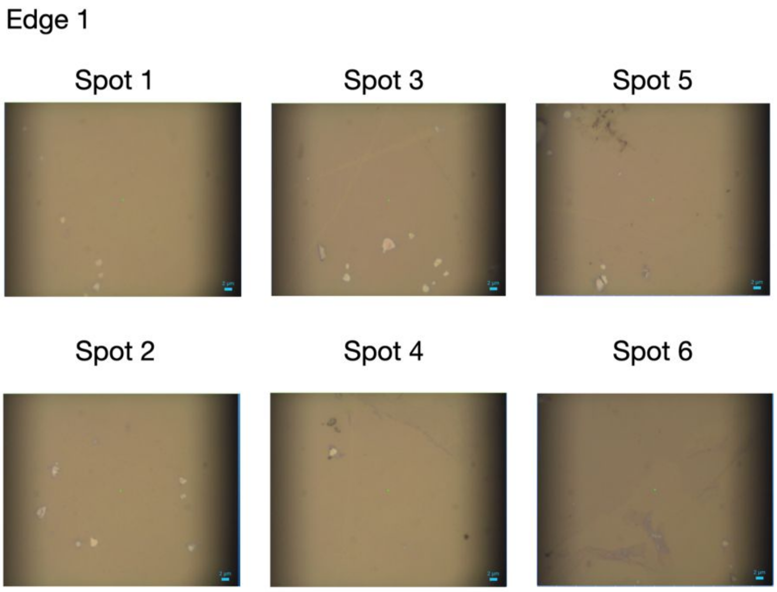

Fig. S6: Optical microscopy image of different spots in the edge 1 region.

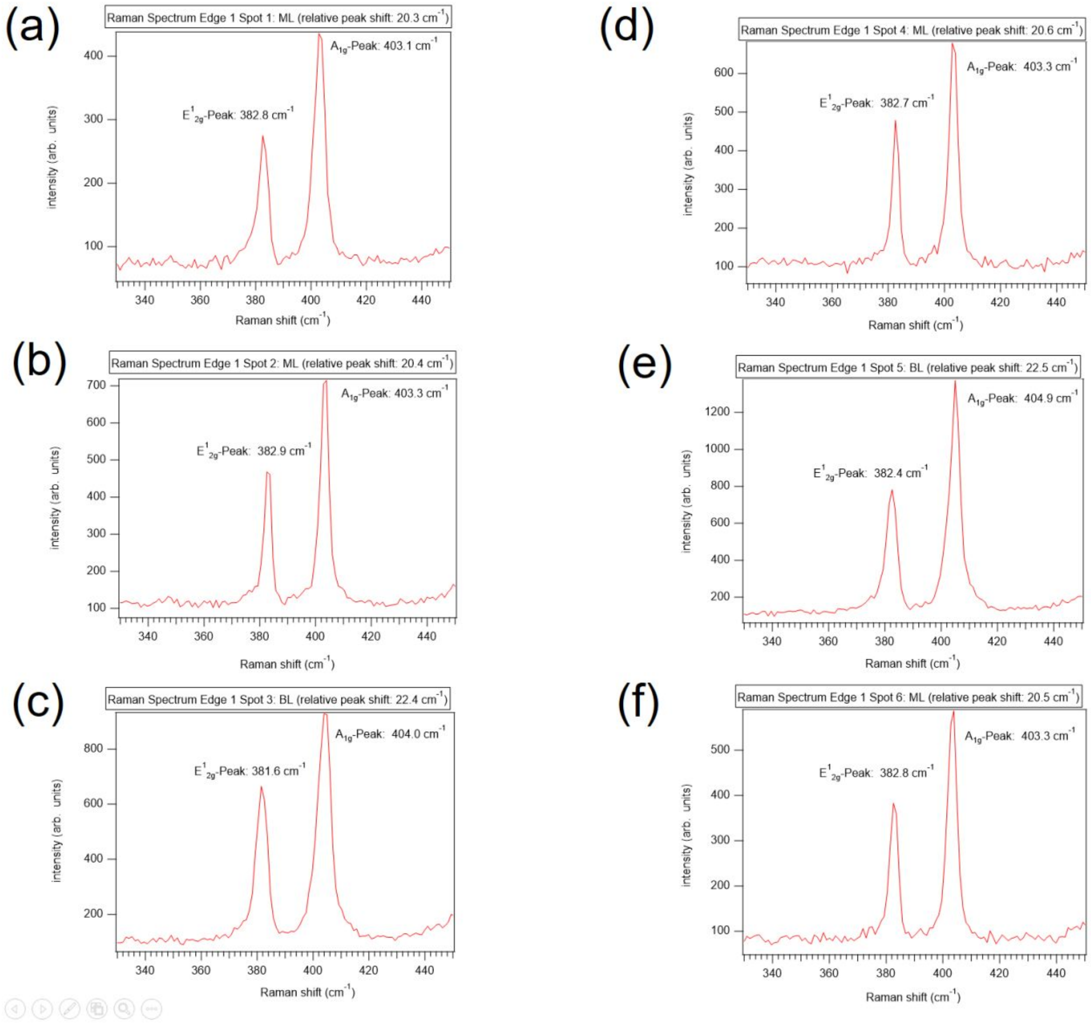

Fig. S7: Raman spectra edge 1 region.

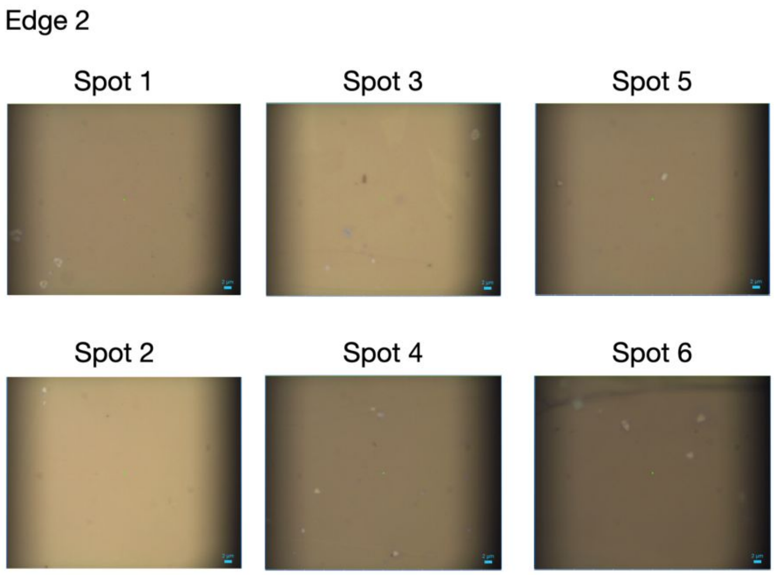

Fig. S8: Optical microscopy image of different spots in the edge 2 region.

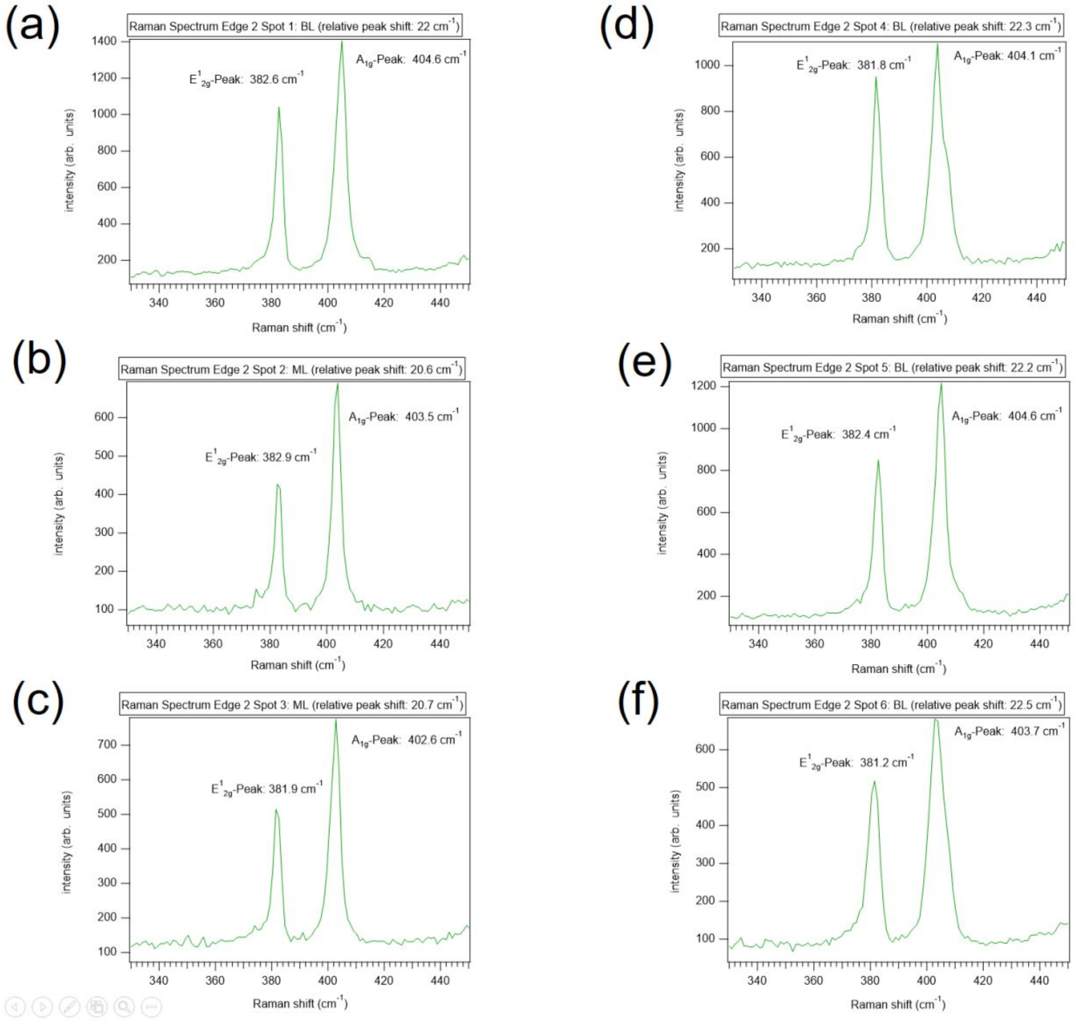

Fig. S9: Raman spectra edge 2 region.

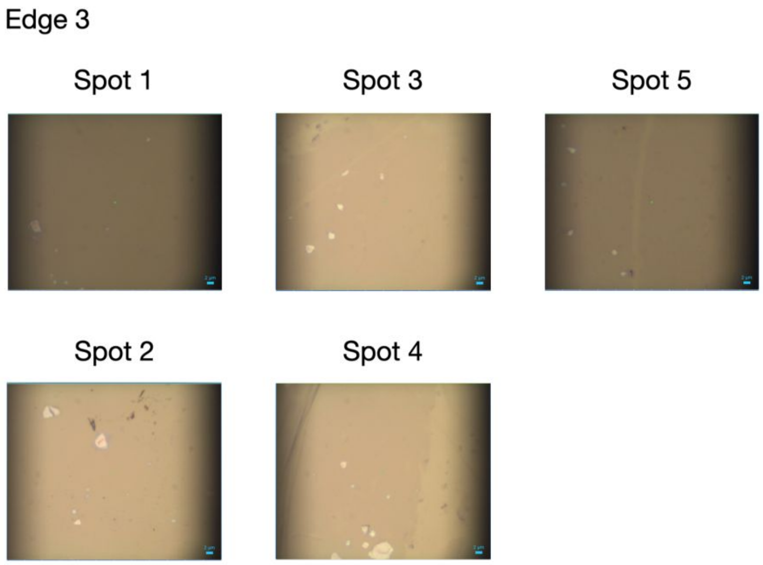

Fig. S10: Optical microscopy image of different spots in the edge 3 region.

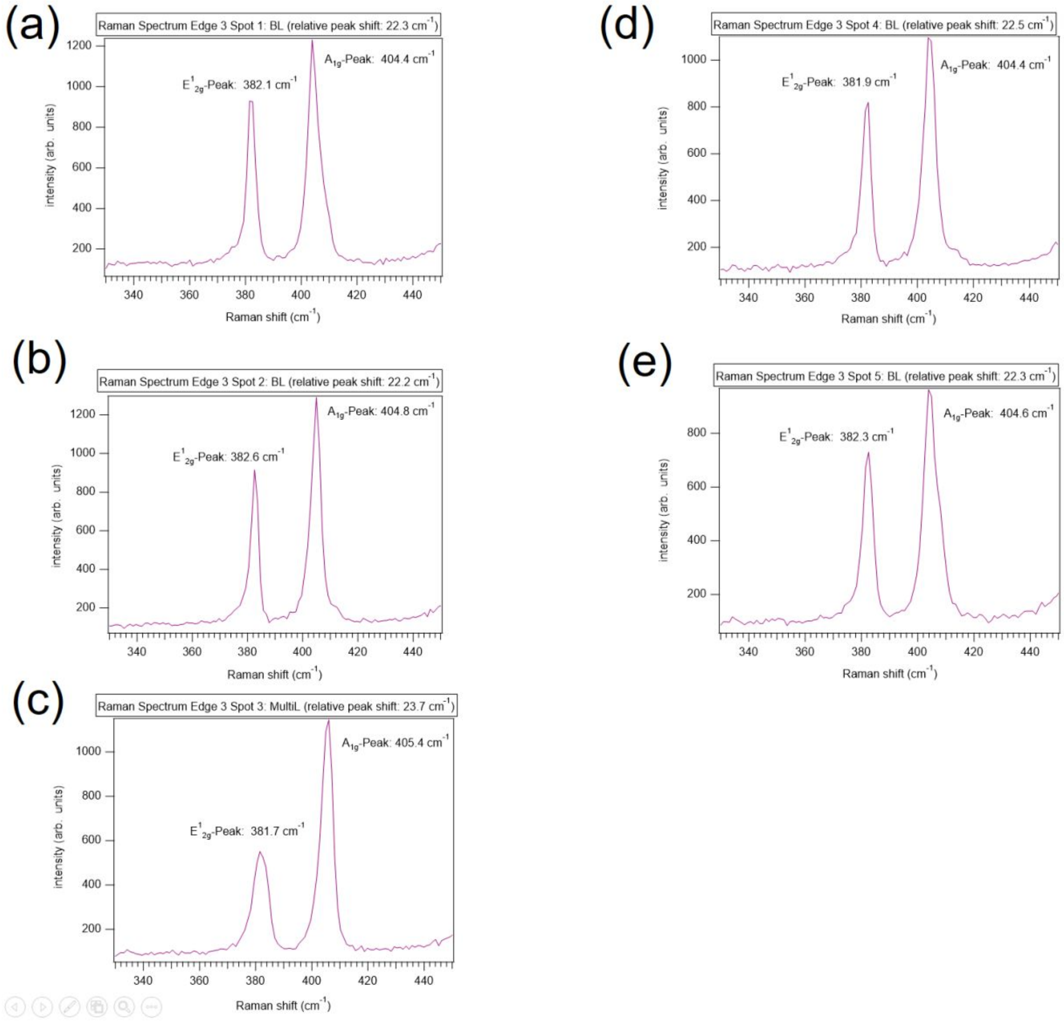

Fig. S11: Raman spectra edge 3 region.

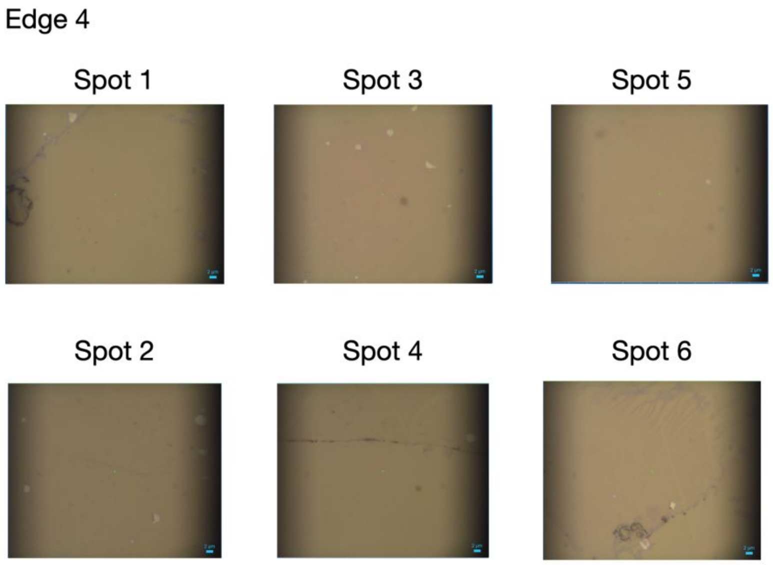

Fig. S12: Optical microscopy image of different spots in the edge 4 region.

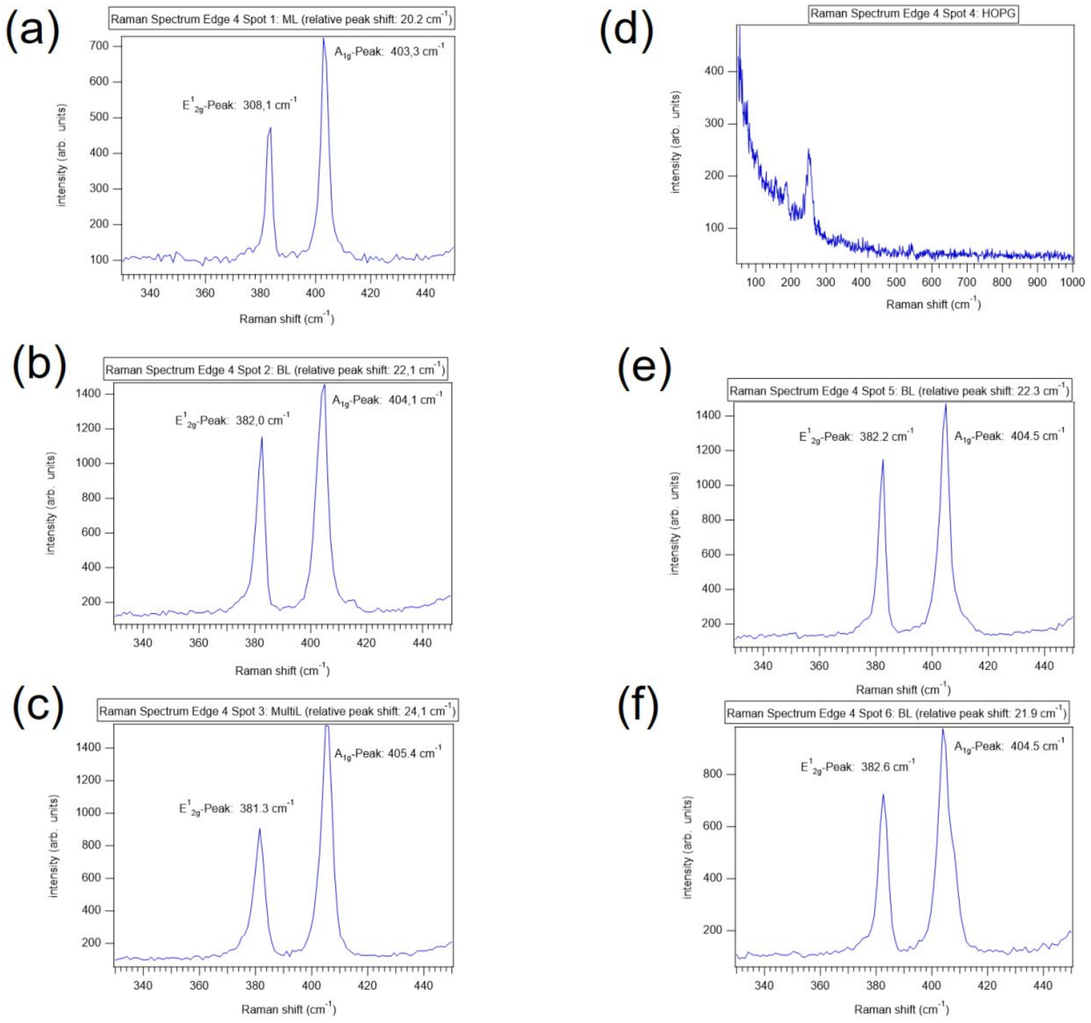

Fig. S13: Raman spectra edge 4 region.

2. *XPS-survey spectra*

For each molecular deposition a survey XPS spectrum was acquired. Fig. S14 shows the XPS survey spectra in the binding energy range from 0 eV to 600 eV. The results for the exemplary depositions of 1.6 Å of the molecular n-type dopant $[RuCp*mes]_2$ as well as for a deposition of 0.5 Å of the molecular p-type dopant F_6TCNNQ are shown in Fig. S15. Additionally, through AR-XPS measurements the deposition of the respective molecular dopant was once more checked in the region of Ru 3d and F 1s core levels.

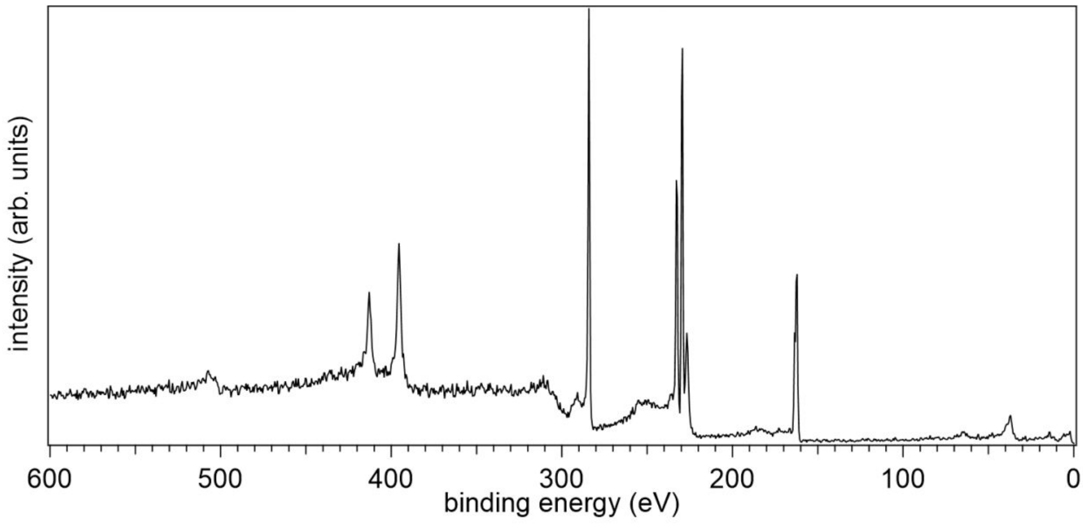

Fig. S14: XPS survey spectra of clean BL-MoS_2.

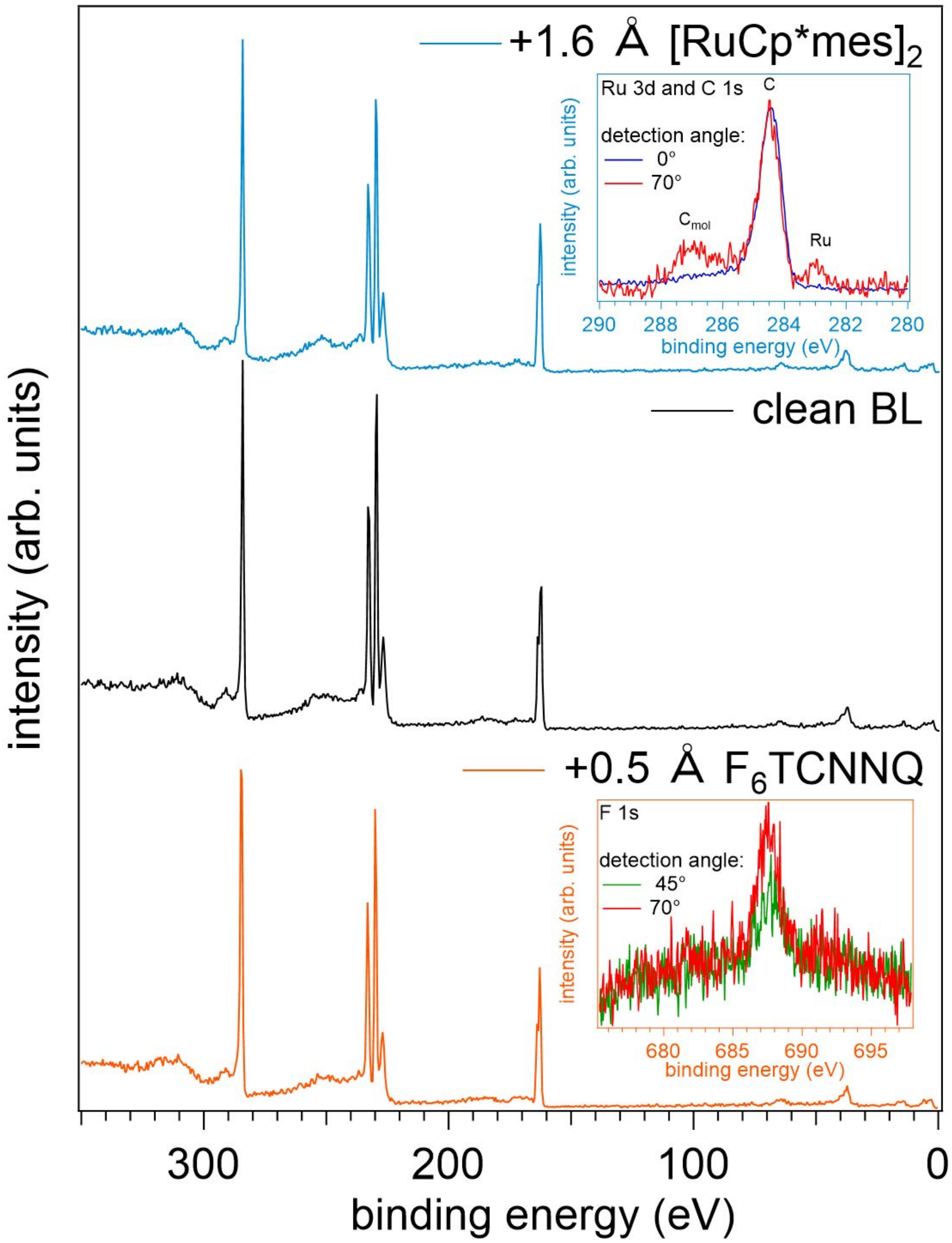

Fig. S15: XPS survey spectra of clean BL and upon the molecular deposition of 0.5 Å F_6TCNNQ and 1.6 Å [RuCp*mes]$_2$, and respective AR-XPS spectra in core level region of the single molecular constituents.

3. *Molecular dopants*

In the following the chemical structure of the two molecular dopants: p-type donor F_6TCNNQ and n-type donor $[RuCp^*mes]_2$ is given[10,11].

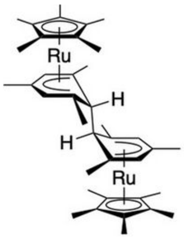

Fig. S16: Chemical structure of the molecular n-type donor $[RuCp^*mes]_2$.[10]

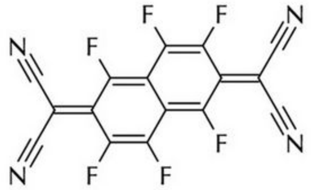

Fig. S17: Chemical structure of the molecular p-type donor F_6TCNNQ.[11]

4. *Inelastic mean free path quantification*

For a quantitative analysis of the core level peaks obtained from XPS measurements, the inelastic mean free path (IMFP) of electrons in the kinetic energy (E_{KIN}) range corresponding to the core levels needed to be determined, as the attenuation follows the Lambert-Beer law. For this aim, the carbon 1s signal from a clean HOPG substrate was then compared to the same signal after deposition of BL-MoS_2.

The fitting results yielded an area under the curve of the C 1s peak of 604.2 for clean HOPG and 368.5 after the transfer of BL-$MoS_{2,}$ with a measurement uncertainty of 1%. This corresponds to an attenuation of 61% of the C 1s signal due to the BL-TMDC. A schematic diagram of the performed measurements is shown in Fig. S18. Using the Lambert-Beer law, this attenuation results in an extrapolated IMFP of (28±1) Å. Notably, a change in the IMFP by ±1 Å only affects the attenuation factor by 1%. A total thickness of 14 Å of the BL-MoS_2 was assumed for the calculation.

It was found that at an emission angle of 0° (normal emission mode), the bottom component is attenuated by (78.0±0.5)% by the surface component. At an emission angle of 70° (grazing emission mode), the attenuation caused by the surface components reduces the signal of the bottom component by (49.0±0.5)% compared to the surface component signal.

With an IMFP of around 28 Å and attenuation factors of approximately 0.78 and 0.49 in normal and grazing emission modes, respectively, for the bottom component relative to the surface component, a complete quantification of the XPS data can be achieved.

Although these values are specifically related to the E_{KIN} range of the electrons associated with the binding energy of the C 1s peak, the results remain reliable for other core level peaks under analysis,

as the binding energy shifts within 130 eV, which does not significantly alter the IMFP on the universal curve.

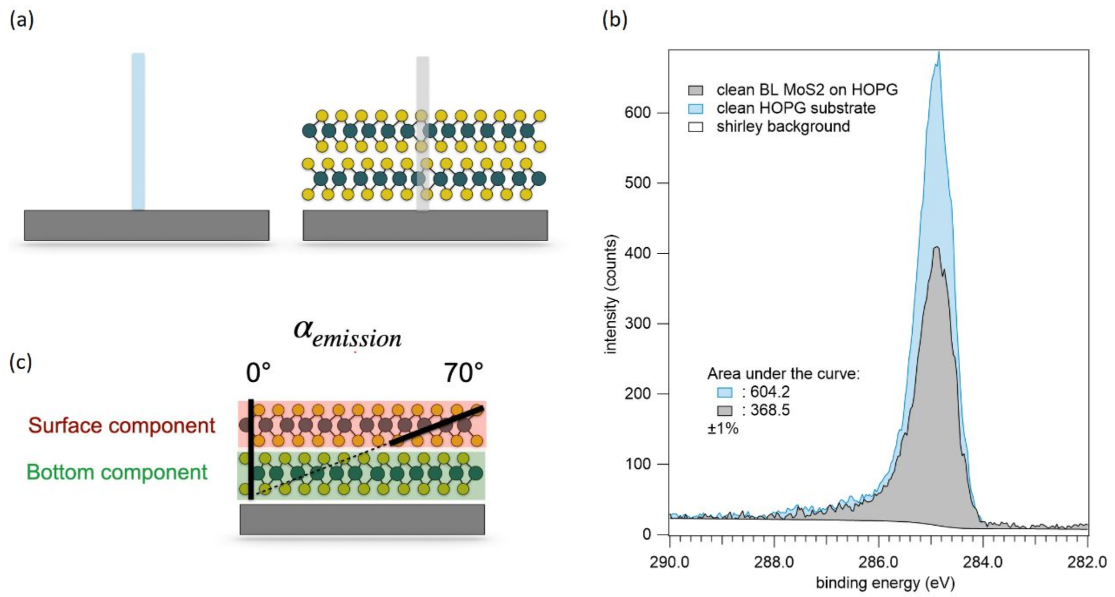

Fig. S18: XPS measurements performed for the quantification of the IMFP of electrons with the kinetic energy associated to the C 1s peak. SubFig. (a) shows a schematic representation of the performed XPS measurements for the C 1s core level area analysis of clean HOPG (blue) and BL-MOS_2/HOPG (grey). The respective experimentally found C 1s core level data is shown in subFig. (b), with found values of the area under the curve emerging from the fit. SubFig. (c) shows the schematic representation of the surface sensitivity changes for different emission angles in XPS measurement. Both solid black lines have the same length. As the first covers completely both surface (red) and bottom (green) component, the 70° tilted barely manages to cover the surface component (red).

5. Core level analysis, two component model and fitting procedure

The samples underwent angle-resolved X-ray photoelectron spectroscopy (AR-XPS) measurements, in order analyse the influence of the emerged electrical field on the core levels Mo 3d, S 2p and S 2s of the BL-MoS_2. The electrical field is oriented in direction normal to the samples surface. The potential felt by each ML-MoS_2 (surface and bottom) is therefore expected to be different, see Fig. S19a. Three scenarios can now appear for each core level: the differential shift of bottom and surface component completely, partially or doesn't overcome the width of the single core levels, see Fig. S19b (i), (ii), and (iii) respectively. To determine whether this two-component model reflects the real potential landscape, the appearance of scenario (i) and (ii) are sufficient to state the correctness of the model. In the third case (iii), one additional AR-XPS measurement must be performed, shown in Fig. S19c. Modifying the angle of detection of the photo emitted electrons allows tuning the surface sensitivity of the measurement and therefore the ratio of the contribution of the surface and bottom component in the final core level peak. If the peak results shifted towards the surface component for higher angles in comparison to lower for the same molecular deposition, the two-component model is proven.

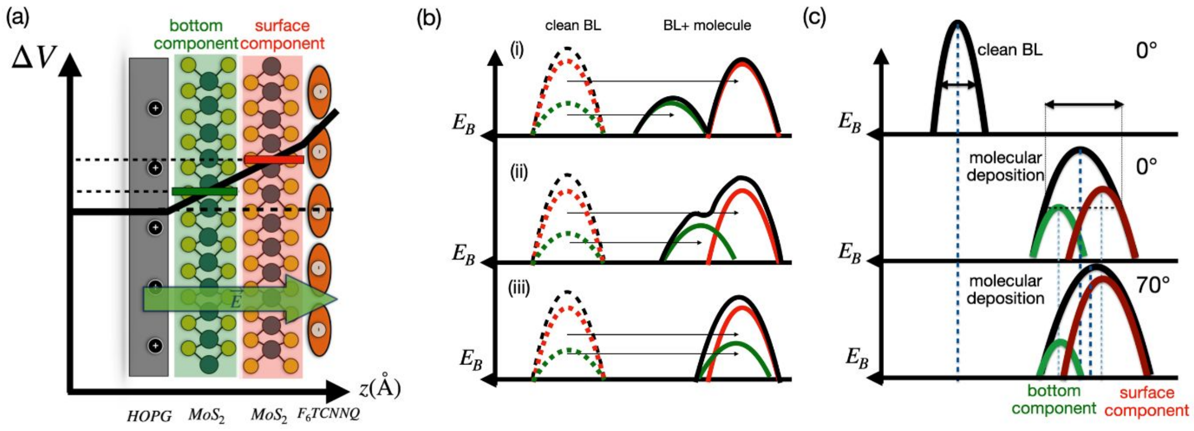

Fig. S19: Schematic potential drop caused by emerged electrical field upon molecular acceptor deposition showing the averaged potential landscape felt by each ML-MoS_2 components (a). Three possible scenarios of expected single core level peak shifts prior and upon molecular deposition for each component (b): bottom and surface component shift with respect to one another for a value above the full width of the single core levels, for each core level in the case of the clean BL, two completely distinguishable peaks emerge upon molecular deposition (i); the differential shift between surface and bottom component upon molecular deposition is below the full width of the single peaks, nevertheless for each core level peak prior to molecular deposition a final core level peak with a strong double component contribution and strong asymmetry is found (ii); the differential shift of bottom and surface component only enables a final core level peak upon molecular deposition with increased broadening in comparison to the clean BL (iii). In case of the scenario shown in (b,iii) increasing the detection angle of the XPS from e.g. 0° to 70° increases the contribution of the surface component in comparison to the bottom component in the final peak: for the same molecular deposition thickness, the final peak is expected to shift towards the surface component (c).

As can be observed from the measurements reported in the main manuscript, the peaks upon molecular deposition broaden and shift towards lower binding energies, as already observed in the valence band (VB), but do not show clear asymmetry or two component features, nevertheless did the AR-XPS measurement proof the correctness of the two-component model.

a. Results of line shape optimization

All XPS measurements underwent a fitting procedure to quantify the energy shifts through the CASA XPS software. First, an optimization of the line shape was performed using a Gaussian-Lorentzian convolution, the final results are shown in Table S2. Generally, an increase in the Lorentzian component and not of the Gaussian component is found if the peaks are fitted with a single component, meaning that the broadening is not solely scattering induced. The line shape of the two-component peaks doesn't significantly alter before and after molecular deposition. Secondly, a Shirley background was subtracted and the theoretically expected shifts between spin splitted peaks constraint. The ratio of surface and bottom component was confined to 0.78 and 0.49 for the emission angle 0° and 70°, respectively. These values were found through the experimental determination of the IMFP of (28 ± 1) Å and application of Beer-Lambert law. For further information please consult the "Inelastic mean free path quantification" of the SI. Finally, for both measurements for the molecular deposition, the binding energy within the same component was constraint to be equal. All energy values carry an uncertainty of 30 meV.

Core level		Mo 3d	S 2s & 2p
Clean BL-MoS_2		GL(70)	GL(61)
0.5 Å F_6TCNNQ	One component	GL(61)	GL(41)
	Two components	GL(74)	GL(62)
Clean BL-MoS_2		GL(67)	GL(33)
1.6 Å $[RuCp^*mes]_2$	One component	GL(63)	GL(33)
	Two components	GL(70)	GL(40)

Table S2: Peak shape optimization results of the Mo 3d, S 2s and S 2p core levels for clean BL-MoS_2 and after deposition of molecular dopants. One component and two components indicates whether the differential shift of the two ML-MoS_2 was neglected or not.

b. Control of electronic stability

The electronic stability of the measurements was checked on the Mo 3d core level peak for the deposition of 1.6 Å of $[RuCp^*mes]_2$ on BL-MoS_2. For this deposition, the first measurement was taken exactly on this core level, after which the complete ARPES, UPS, and AR-XPS characterization followed. At the end of the measurement series, the same core level was measured again. The results are shown in Fig. S20; no shift above the normal sensitivity of 30 meV was detected. The electronic stability of the measurements is therefore considered valid.

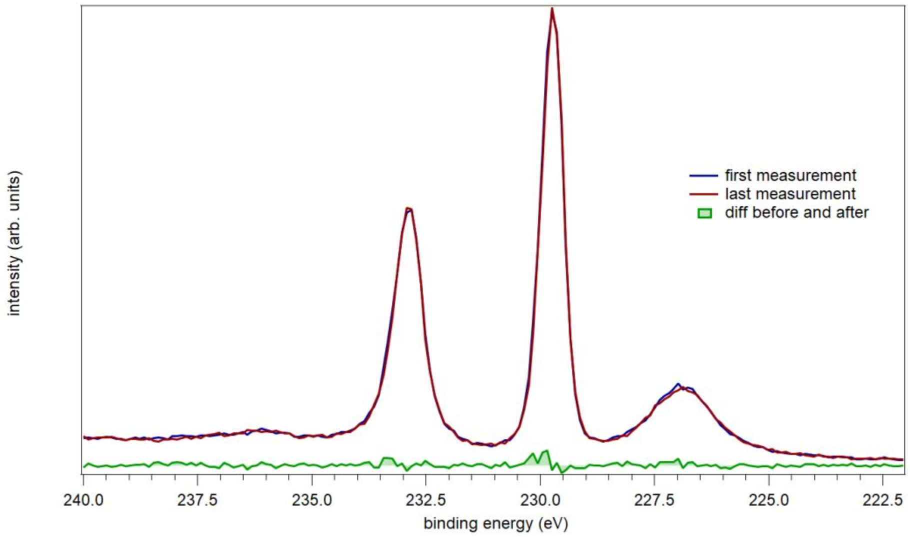

Fig. S20: Mo 3d core level peak of BL-MoS_2 upon deposition of 1.6 Å of $[RuCp^*mes]_2$ as first measurement of sample characterisation of that specific deposition thickness, and as control measurement after the complete characterisation with ARPES, UPS and AR-XPS. Time difference between the measurements around 12 hours.

c. XPS observations other thicknesses

The AR-XPS measurement were performed for different thicknesses of the molecular electron acceptor and donor. The results of the two-component model fitting procedure is shown in Table S3 and Table S4 , respectively.

Peak positions: surface vs bottom layer MoS_2				
	Mo 3d 5/2		S 2p 3/2	
	0°	70°	0°	70°
Clean BL-MoS_2	230.05<br>STD=7.8<br>FWHM: 0.47		162.87<br>STD=8.5<br>FWHM: 0.48	
0.5 Å F_6TCNNQ on BL-MoS_2	229.81 eV (surf)<br>230.96 eV (bottom)<br>STD=8.3<br><br>229.90 eV (one comp)<br>STD=8.3<br>FWHM: 0.54	229.81 eV (surf)<br>229.96 eV (bottom)<br>STD=7.8<br><br>229.83 eV (one comp)<br>STD=7.6	162.63 eV (surf)<br>162.81 eV (bottom)<br>STD=8.7<br><br>162.73 eV (one comp)<br>STD=7.4<br>FWHM: 0.57	162.62 eV (surf)<br>162.80 eV (bottom)<br>STD=8.7<br><br>162.66 eV(one comp)<br>STD=8.8
1.3 Å F_6TCNNQ on BL-MoS_2	229.69 (surf)<br>229.88 (bottom)<br>STD=7.3<br><br>229.76 eV (one comp)<br>STD=7.6	229.69 (surf)<br>229.89 (bottom)<br>STD=7.4<br><br>229.76 eV (one comp)<br>STD=6.8	162.48 eV (surf)<br>162.70 eV bottom)<br>STD=7.3<br><br>162.58 eV (one comp)<br>STD= 8.1	162.55 eV (surf)<br>162.68 eV (bottom)<br>STD=7.1<br><br>162.60 eV (one comp)<br>STD =9.0
29.3 Å F_6TCNNQ on BL-MoS_2	229.63 (surf)<br>229.83 (bottom)<br>STD=6.5<br><br>229.71 (one comp)<br>STD=6.9		162.39 (surf)<br>162.62 (bottom)<br>STD=8.8<br><br>162.48 (one comp)<br>STD=8.4	

Table S3: Fitting parameters and results of the XPS measurements performed on BL-MoS_2 upon deposition of different thicknesses of the molecular p-type dopant F_6TCNNQ.

Peak positions: surface vs bottom layer MoS_2				
	**Mo 3d 5/2**		**S 2p 3/2**	
	**0°**	**70°**	**0°**	**70°**
Clean BL-MoS_2	229.75 eV<br>STD=17.4<br>FWHM: 0.53		162.54 eV<br>STD=9.8<br>FWHM: 0.57	
0.8 Å $[RuCp^*mes]_2$ on BL-MoS_2	229.95 eV (surf)<br>229.73 eV (bottom)<br>STD= 9.5		162.80 eV (surf)<br>162.58 eV (bottom)<br>STD=14.6	
1.6 Å $[RuCp^*mes]_2$ on BL-MoS_2	229.89 eV (surf)<br>229.76 eV (bottom)<br>STD=11.8<br>229.83 eV (one comp)<br>STD=9.5<br>FWHM:0.63	229.91 eV (surf)<br>229.76 eV (bottom)<br>STD=14.3<br>229.88 eV (one comp)<br>STD=9.9	162.81 eV (surf)<br>162.60 eV (bottom)<br>STD=7.7<br>162.70 eV (one comp)<br>STD=8.3<br>FWHM:0.63	162.83 eV (surf)<br>162.64 eV (bottom)<br>STD=8.1<br>162.76 eV (one component)<br>STD=9.9
13.2 Å $[RuCp^*mes]_2$ on BL-MoS_2	229.90 eV (surf)<br>229.66 eV (bottom)<br>STD=13.5		162.73 eV (surf)<br>162.58 eV (bottom)<br>STD=15.0	

Table S4: Fitting parameters and results of the XPS measurements performed on BL-MoS_2 upon deposition of different thicknesses of the molecular p-type dopant $[RuCp^*mes]_2$.

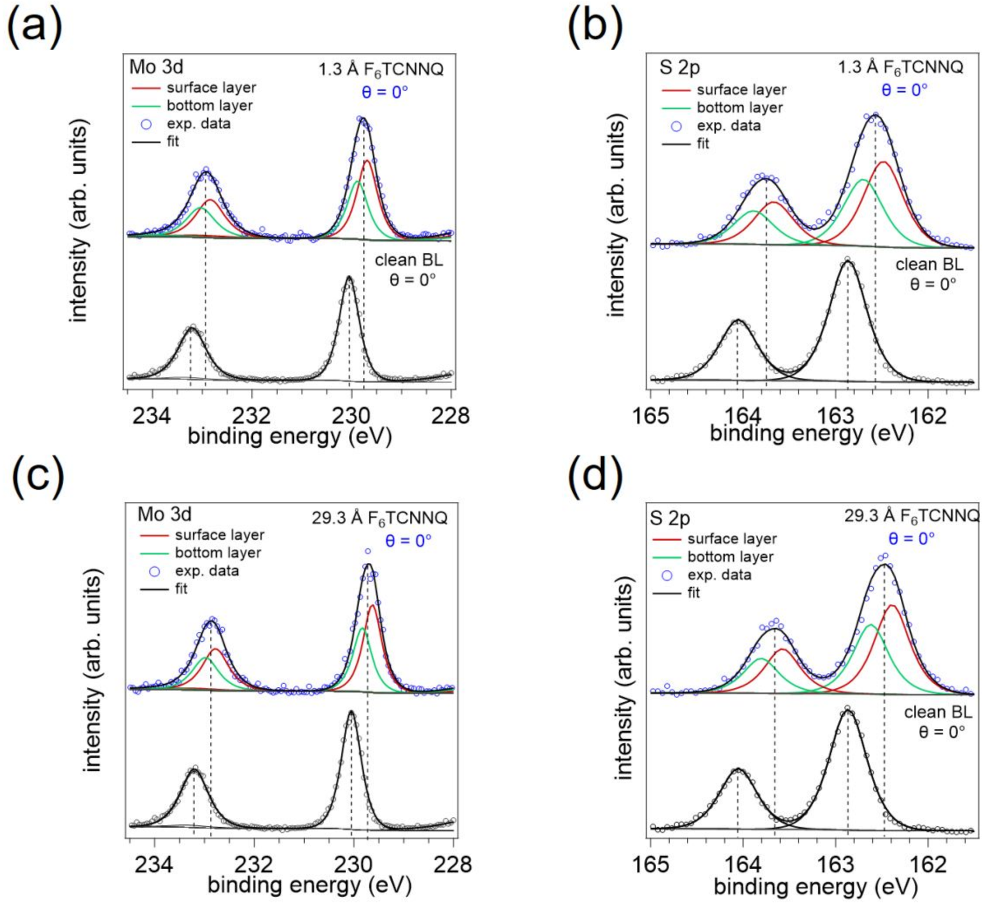

Fig. S21: XPS fitting of Mo 3d and S 2p peak of XPS measurements performed on BL-MoS_2 upon deposition of different thicknesses of the molecular p-type dopant F_6TCNNQ.

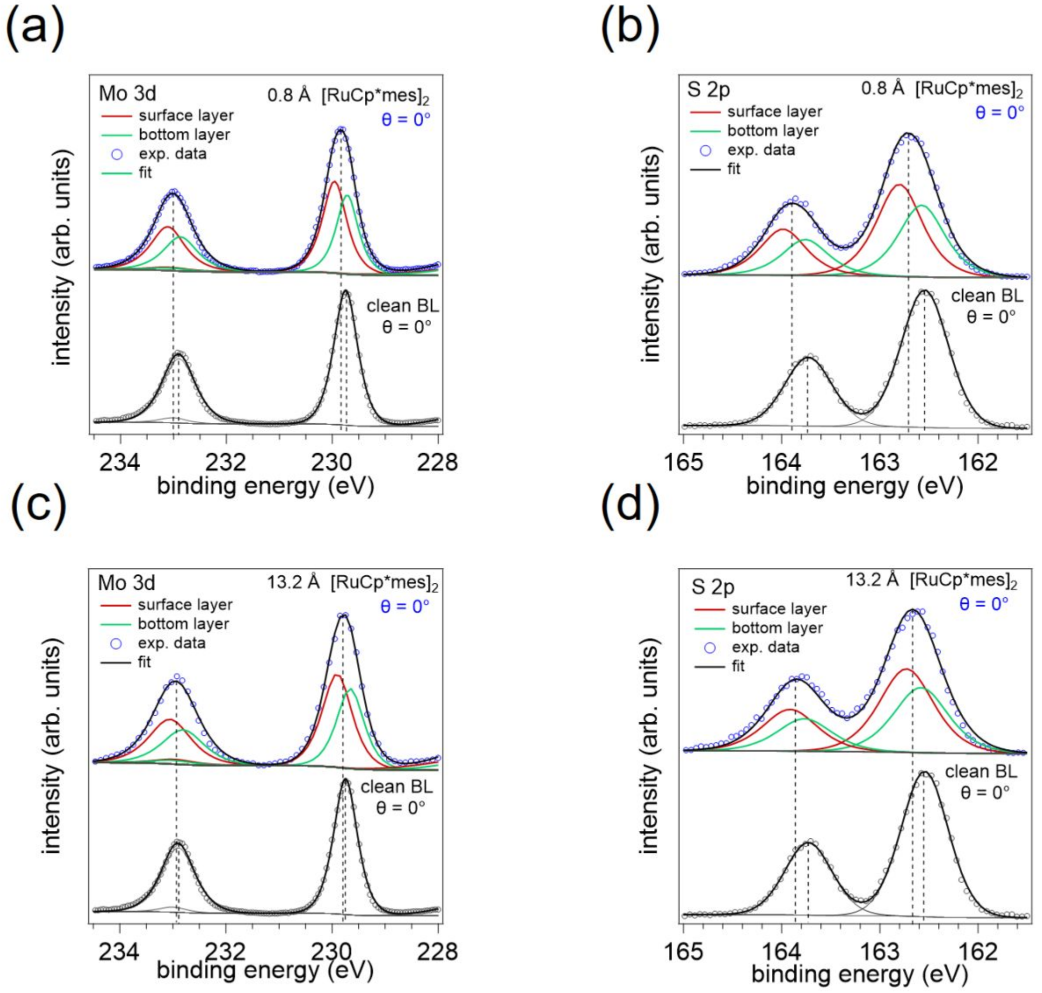

Fig. S22: XPS fitting of Mo 3d and S 2p peak of XPS measurements performed on BL-MoS_2 upon deposition of different thicknesses of the molecular n-type dopant [RuCp*mes]$_2$.

6. *Band structure observations other thicknesses*

a. *p-type dopant*

Valence band upshift

The characterization of the valence band (VB) upon deposition of the molecular p-type dopant F_6TCNNQ were repeated for different deposition thicknesses, Table S5 summarizes the found values of the different bands for different layer thicknesses, while Fig. S23 and Fig. S24 show the measured band structure around the K- and Γ-point.

Molecule	Total deposition thickness	Local VBM at K-point (spin splitted bands)	Global VBM at Γ-point (peak value ± FWHM of EDC)	
	(Å)	K_1	Γ_1	Γ_2

BL-MoS_2		1.82	2.03	1.58 $\pm$ 0.24	2.10 $\pm$ 0.16
F_6TCNNQ	0.5	~1.62		1.43 $\pm$ 0.27	1.97 $\pm$ 0.25
	1.3	~1.55		1.33 $\pm$ 0.30	1.89 $\pm$ 0.30
	29.3	~1.53*		1.28 $\pm$ 0.27	1.87 $\pm$ 0.27
All values in eV<br>Error: $\pm$ 30 meV					

Table S5: Binding energy values of global and local VBM of BL-MoS_2 before and after deposition of molecular acceptor layer.

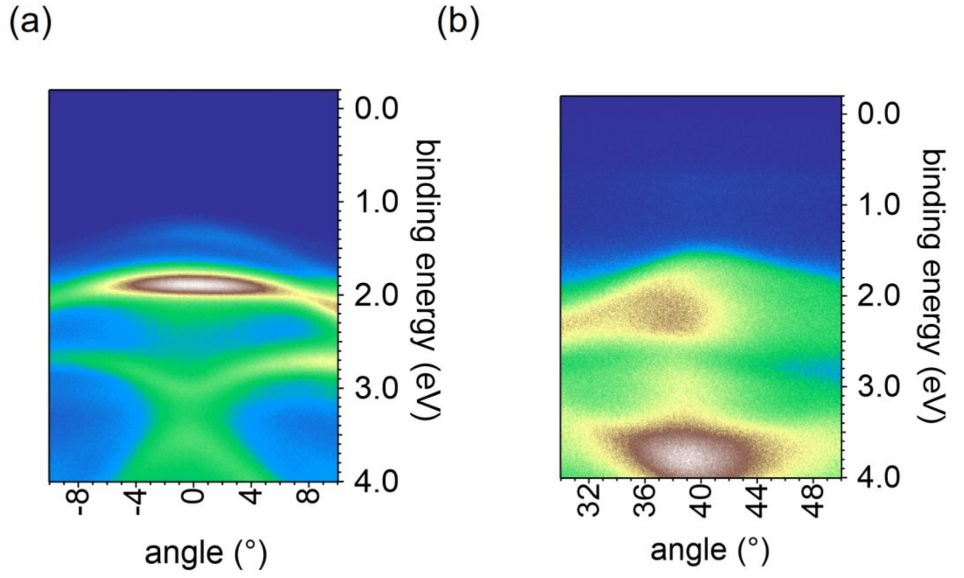

Fig. S23: ARPES spectrum of band dispersion at Γ-point (a) and K-point (b) of BL-MoS_2 after deposition of molecular p-type acceptor (1.3 Å F_6TCNNQ).

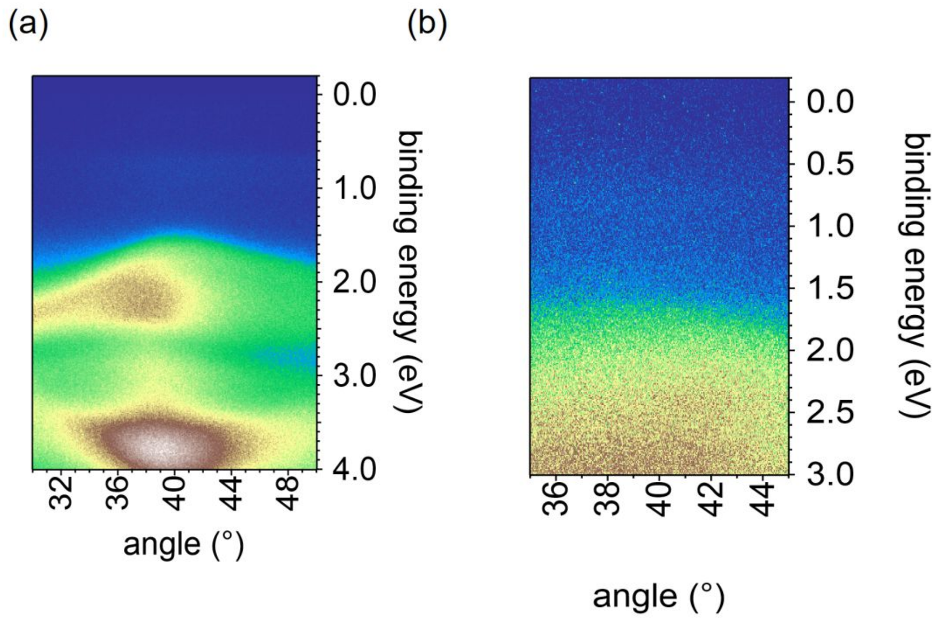

Fig. S24: ARPES spectrum of band dispersion at Γ-point (a) and K-point (b) of BL-MoS_2 after deposition of molecular p-type acceptor (29.3 Å F_6TCNNQ).

b. *n-type dopant*

VB downshift

The characterization of the VB upon deposition of the molecular n-type dopant $[RuCp^*mes]_2$ was repeated for different deposition thicknesses, Table S6 summarizes the found values of the different bands for different layer thicknesses, while Fig. S25 and Fig. S26 show the measured band structure around the K- and Γ-point.

Upon deposition of 13.2 Å, no band structure was recognizable anymore, due to the strong scattering of the photo emitted electrons with the molecular layer.

Molecule	Total deposition thickness	Local VBM at K-point (spin splitted bands)		Global VBM at Γ-point (peak value ± FWHM of EDC)	
	(Å)	K_1		Γ_1	Γ_2
BL-MoS_2		1.70	1.89	1.58 ± 0.21	2.10 ± 0.17
$[RuCp^*mes]_2$	0.8	~1.86		1.62 ± 0.22	2.20 ± 0.31
	1.6	~1.91		1.64 ± 0.24	2.21 ± 0.33
	13.2	/		/	
All values in eV<br>Error: ± 30 meV					

Table S6: Binding energy values of global and local VBM of BL-MoS_2 before and after deposition of molecular donor layer.

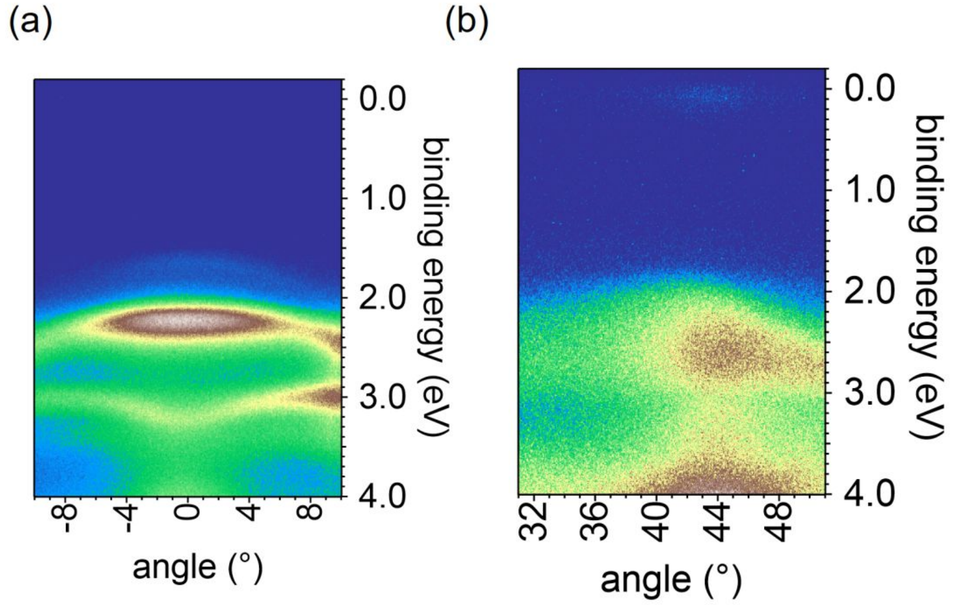

Fig. S25: ARPES spectrum of band dispersion at Γ-point (a) and K-point (b) of BL-MoS_2 after deposition of molecular n-type donor 0.8 Å [RuCp*mes]$_2$.

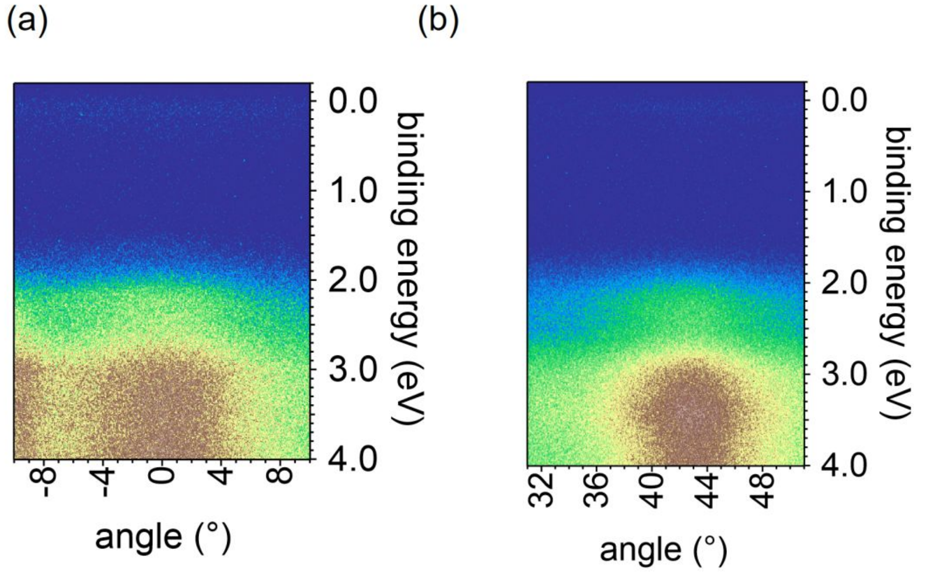

Fig. S26: ARPES spectrum of band dispersion at Γ-point (a) and K-point (b) of BL-MoS_2 after deposition of molecular n-type donor 13.2 Å [RuCp*mes]$_2$.

Q-point

Fig. S27 shows the ARPES spectra for different deposition thicknesses of the molecular n-type dopant [RuCp*mes]$_2$ around the Γ and Q point. As can be clearly observed no increase of the DOS around the Fermi level (E_F) is observed for the first two depositions (0.8 Å (b) and 1.6 Å (c)), while it was observed around the K-point. This leads to the conclusion that the conduction band minimum (CBM) sits at the reciprocal lattice point K. The increased DOS at the highest deposition (13.2 Å (d)) is

constant over the whole momentum range from the Γ to the Q point and is therefore caused by the increased scattering of the electrons with the thick molecular layer.

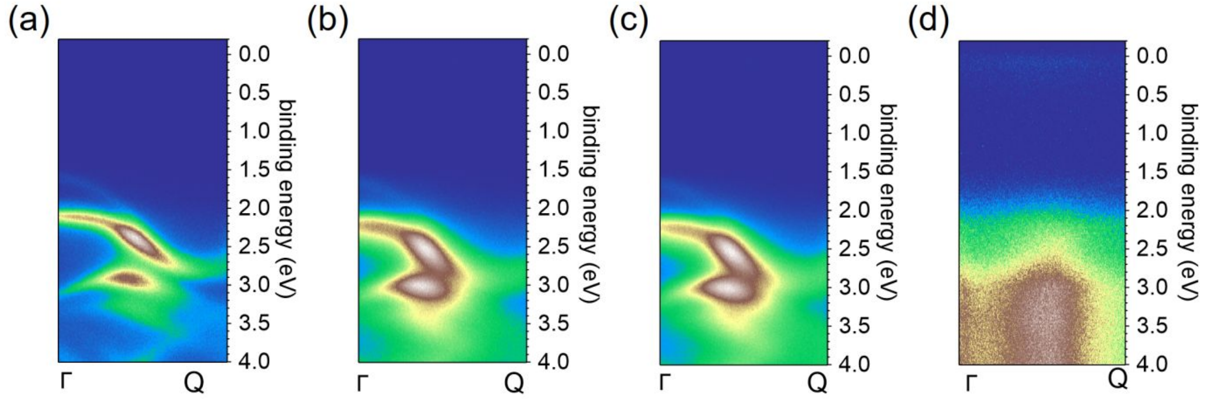

Fig. S27: ARPES spectra around the reciprocal lattice point Γ and Q of the clean (a) BL-MoS_2 and upon deposition of 0.8 Å (b), 1.6 Å (c) and 13.2 Å (d) of the molecular n-type dopant $[RuCp^*mes]_2$.

7. Relation of ARPES and XPS observations: reconstruction of the K-point (summation)

The CBM and VBM of BL-MoS_2 are completely defined by the Mo d-orbitals. While at the global VBM, the Γ-point is defined by the Mo out-of-plane d_{z^2} orbitals, the local VBM at the K-point is defined by the Mo in plane d_{xy,x^2-y^2} orbitals[12]. This means that the VB at K shows a direct reflection of the locally felt potential within each layer. Therefore, it should be possible to reconstruct the EDC around K upon molecular deposition with two EDC curves of the clean BL resembling the surface and bottom component, each integrated over a broader range to mimic scattering by the molecular layer, shifted accordingly to the observations made during the XPS measurements and attenuated in respect to one another as expected from this surface sensitive technique. The results are shown in the main paper for the deposition of 0.5 Å of the molecular p-type acceptor molecule F_6TCNNQ and 1.6 Å of the molecular n-type donor molecule $[RuCp^*mes]_2$. A perfect overlay of the experimental and reconstructed EDCs is observed, stating the integrity and correlation of the two measurement techniques. Small deviations are found for binding energies above 2.50 eV, where additionally also the sulfur orbitals compose the VB of BL-MoS_2. In the following we present the results found through the same procedure, found for different deposition thicknesses than the one presented in the paper. Also here the results show the reproducibility of the technique and consistency of the model.

for molecular acceptor

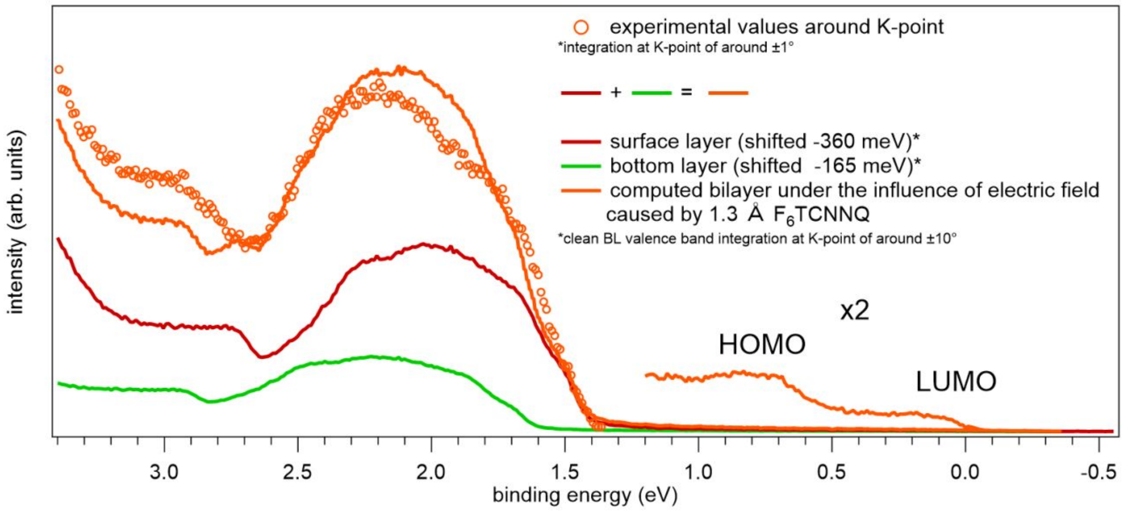

Fig. S28: EDC reconstruction of K-point upon deposition of 1.3 Å F_6TCNNQ on BL- MoS_2 reconstructed through the sum of two shifted and scales EDC of the clean BL system.

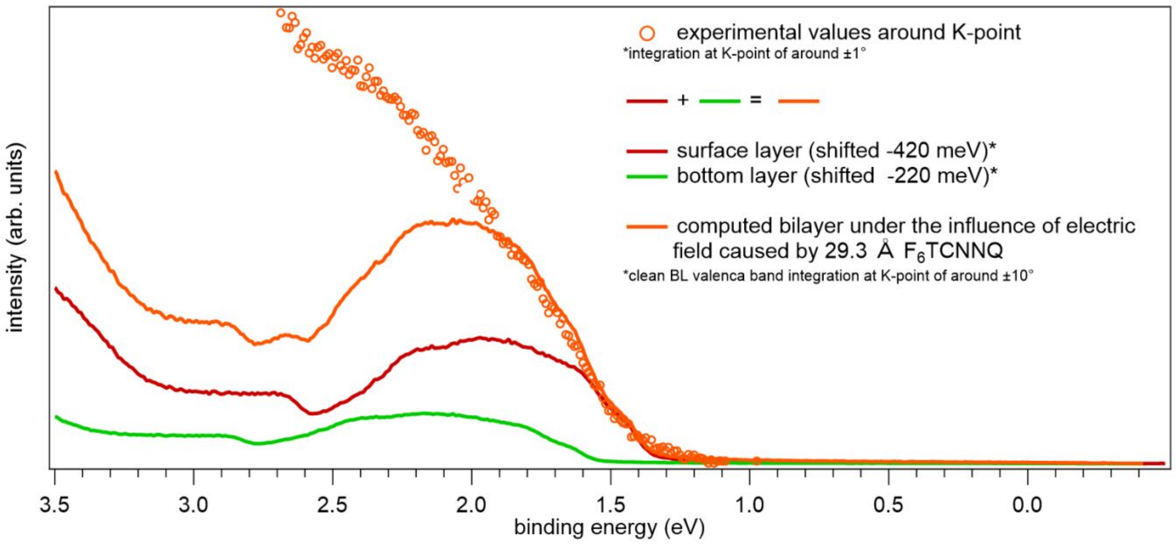

Fig. S29: EDC reconstruction of K-point upon deposition of 29.3 Å F_6TCNNQ on BL-MoS_2 reconstructed through the sum of two shifted and scales EDC of the clean BL system.

for molecular donor

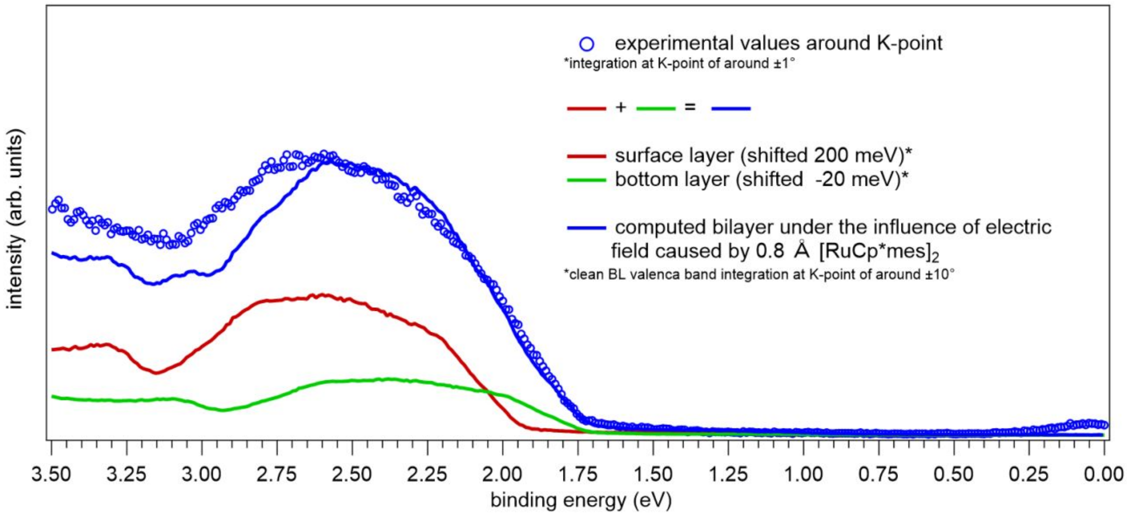

Fig. S30: EDC reconstruction of K-point upon deposition of 0.8 Å $[RuCp*mes]_2$ on BL-MoS_2 reconstructed through the sum of two shifted and scales EDC of the clean BL system.

Reconstruction through summation approach of Γ-point

The used approach of reconstructing the EDC curves around the K-point of the reciprocal lattice works as the molybdenum orbitals that define the VB in this specific point are lying in plane. They therefore are a direct measure of the local potential within the layer. On the other hand, the bands defined around the Γ-point extend in the out-of-plane direction. They therefore are under the influence of a range of the potential profile of the sample, which would not only result in two shifted peaks as at K-point but also in their broadening[12]. This is figuratively shown in Fig. S31 (a) and (b) where the agreement between the summation approach and the experimental spectrum is relatively good but the high binding energy tail broadening is missing. Therefore, a reconstruction through the simple summation approach as shown in this chapter, though possible, may not represent the optimal approach. Nevertheless in Fig. S31 (c) the results are shown in the case of the molecular donor deposition.

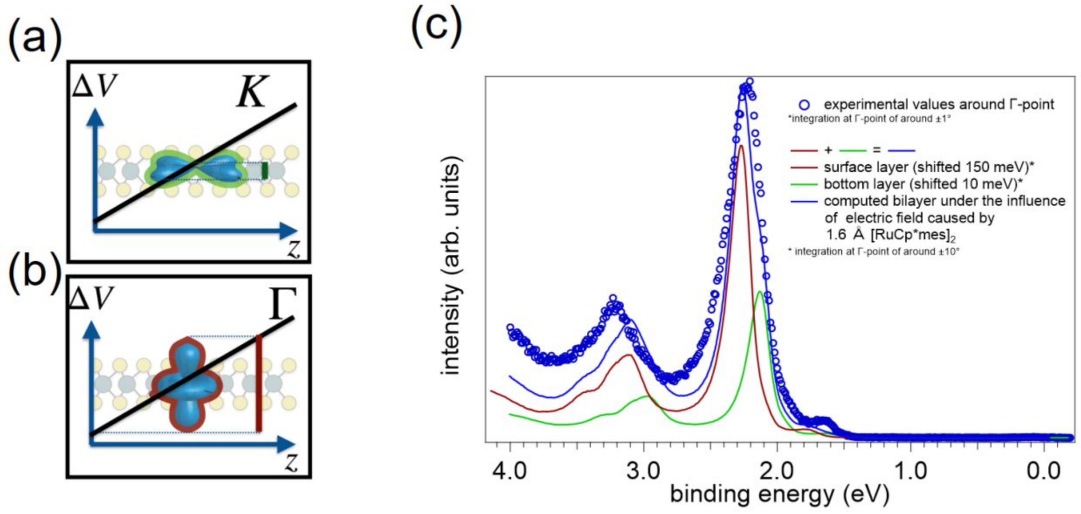

Fig. S31: Schematic description of potential range felt by the in plane Mo d_{xy,x^2-y^2}-orbitals around the K point (a) and the out of plane Mo d_{z^2}-orbitals. Spatial EDC reconstruction of Γ-point upon deposition of 1.6 Å $[RuCp^*mes]_2$ on BL-MoS_2 reconstructed through the sum of two shifted and scales EDC of the clean BL system (c).

8. Hole effective mass estimation

The valence band maximum (VBM) of the Γ-point represents the global VBM of the band structure of MoS_2. Therefore, in the nearest vicinity of this point, the energetically highest band can be described, in a first approximation by a parabolic dispersion like the one of free particles $\mathrm{E} = \mathrm{E}_0 - \frac{\hbar^2\mathbf{k}^2}{2\mathrm{m}_0}$. This dispersion is well represented for a hole effective mass of 1, as has been overlaid to the curvature plots in Fig. S32. The energy offset E_0 is equal to the value given in Table S5 and Table S6 of the respective deposition of the Γ_1 band.

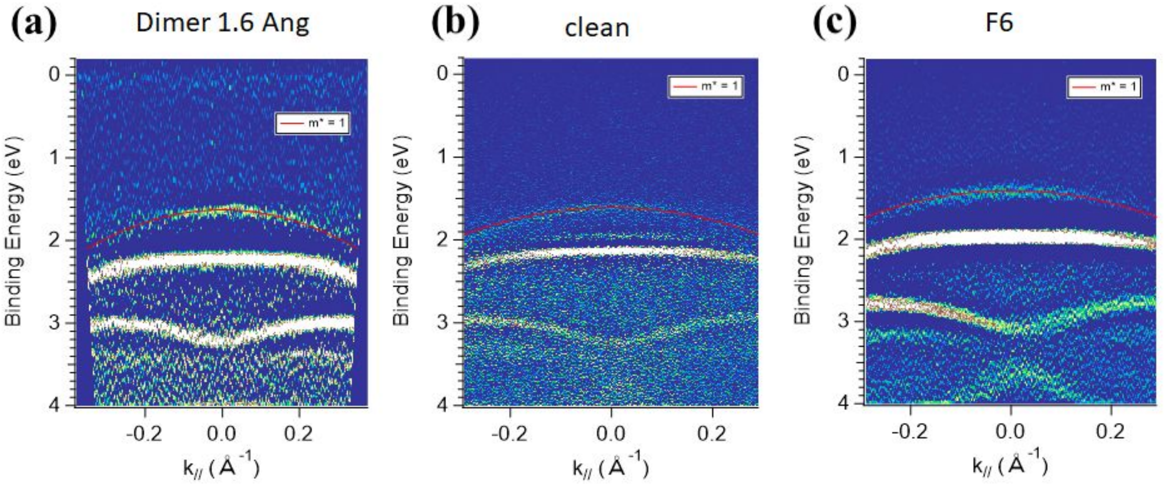

Fig. S32: curvature plots of ARPES spectra around the Γ-point of clean BL-MoS_2 (b) and upon deposition of 1.6 Å of the molecular n-type dopant $[RuCp^*mes]_2$ (a) as well as for a deposition of 0.5 Å of the molecular p-type dopant F_6TCNNQ (c) and fitted free particle energy-momentum distribution for a hole-effective mass of value 1.

9. *Reconstruction of the Γ-point: semiclassical approach to determine the potential landscape*

In the reconstruction of the VB around the K-point performed through the summation approach, the potential change within each layer was approximated to the potential felt by each of the molybdenum layers, as determined from XPS. This is basically equivalent to a convolution operation between the EDC of the clean BL-MoS_2 with two δ-functions set at two different energies corresponding to the XPS shifts.

A more refined picture of the potential landscape can be achieved by reconstructing the details of the EDC around the Γ-point. At the global VBM, in fact, the bands are defined by the out of plane d_{z^2}-orbitals, which therefore feel the continuous change of the potential through the BL[12]. A reconstruction of the EDC curve, therefore, is not as straightforward as it was for the case of the reciprocal lattice point K. Nevertheless, a reconstruction can be performed through a semi classical approach assuming a classical electric field felt by the atomic orbitals. By convoluting the EDC of the clean BL with a function that correlate with the potential evolution in the z-direction, the optimal potential landscape can be determined when a perfect overlap is found between the experimental spectrum of the molecule/BL MoS_2 spectrum and the simulated spectrum. This is similar to what was done at K-point but we then here use a continuous function instead of using two δ-functions to represent the potential affecting the states.

The results of this procedure are shown in the main manuscript, in which the EDC curve before and after a molecular layer deposition are exactly related by this convolution operation. As we propose, this procedure enables the detailed reconstruction of the potential landscape within the BL and, by extrapolation according to the boundary conditions at the HOPG and molecule interfaces, beyond the inorganic-organic and inorganic-semimetal interface regions.

In this section of the SI, the convolution operation of a Gaussian function with different model potential functions is shown in order to make clear the procedure we used.

In Fig. S33, the influence of four differently evolving potential landscapes on an initially grounded state, defined by a Gaussian function centred around an energy of 0 ($gauss_0$), is shown. Different potential profiles pot_{iii} are shown in subfigures (iii)(right column). From this, we then find the function that is used for the convolution product, as a renormalized inverse gradient of the potential ($invgrad_i$), shown for each case respectively in column (i), (left column). The convolution operation results $conv_{ii}$ is shown next to the original Gaussian function in column (ii) middle column.

The convolution function: (f*g)(x)=∫f(x)·g(x-τ)dx applied to our case would then be: $conv_{ii}$(E)= ∫ $gauss_0$(E)· $invgrad_i$(E-τ). G(x) represents the DOS of the system before applying the electric field, f(x) is the convolution kernel that relates to $\left(\frac{dV}{dz}\right)^{-1}$ as mentioned in the main text. (f*g)(x) correspond to the DOS when applying the electric field.

To understand why f(x) correspond to $\left(\frac{dV}{dz}\right)^{-1}$, we now stepwise explain the idea and we neglect any issue related with attenuation of the signal as in photoemission.

1. Fig. S33.a) When the potential is increased by 100 V throughout the sample, the equivalent function for the convolution is a delta function centred at 100 V. The convolution product of this delta function with a Gaussian function (representing the DOS of the sample) is then a Gaussian function shifted by 100 V (case (a)).

2. Fig. S33.b) When the potential linearly increases from 0 to 100 V throughout the sample, the derivative $\frac{dV}{dz}$ is equal to 1/nm. The function f(x) is a step function ranging from 0 to 100 V with magnitude 1nm/V. The convolution show a DOS shifted by 50 V (half the value of the potential drop) and present a broadening of 100 V (case (b.ii)).

3. Fig. S33.c) For a hypothetical 75 nm thick sample, let us consider a potential that increases in the first third (25 nm) of the sample by 2 V/nm (from 0 to 50 V) and by 1V/nm (from 50 to 100V) in the remaining two third of the sample. This means that the initial (gaussian) energy distribution for a flat potential will become two gaussian distributions upon applying this potential: one centered at 25 V and the other centered at 75V. Moreover, as the distribution centered at 25 V corresponds to 1/3 of the sample, its magnitude will be half that of the second gaussian distribution. The effect of such a potential can be represented by the convolution kernel f(x) shown in Fig S33.c.i). The first part of f(x) goes from 0 to 50 with a magnitude of 0.5 and the second part goes from 50 to 100 V with a magnitude of 1. When applying the convolution to a Gaussian distribution, this results in two Gaussian distribution shifted by 25 and 75 V respectively, and a area ratio of 1:2. We note that f(x) is equal to $\left(\frac{dV}{dz}\right)^{-1}$.

4. Fig. S33.d) An analogous example is shown in (d) where the convolution function shows a first step function with magnitude 1 for a potential change from 0 and 50 V and a second step function with magnitude 0.2 for a potential change from 50 to 100 V. The result of the convolution product is then the sum of two convoluted Gaussian, the first shifted by 25 V with a magnitude of 1 and the second is shifted by 75 V and has a magnitude of 0.2. This means that the first part of the potential drop acts on 5/6 of the probed volume while the second part act on 1/6 of the probed volume. This means that the slope of the potential profile is 1 and 5 in the lower and upper parts, respectively. We then can see that the magnitude of f(x) represents $\left(\frac{dV}{dz}\right)^{-1}$.

5. Therefore, by finding the appropriate convolution kernel f(x) with which we can reproduce the molecule-covered EDC from the pristine MoS_2 EDC . Then we can easily track back the potential in real space along the z-direction at the condition that the potential varies monotonously.

6. Furthermore, it is possible to account for the effect of the IMFP by renormalizing z afterwards by the relative signal attenuation.

In summary, applying a constant potential induces a shift in energy of the DOS-distribution by exactly the value of the applied potential. When applying a linearly increasing potential, [case (b)], a broadening in energy of the electronic states distribution is found equal to the applied potential difference and the shift is half the magnitude of the potential change. In case a the potential gradient varies over the sample thickness, an asymmetrical broadening should emerge. In the present examples c and d, this is seen through atwo-component distribution. The strength of the asymmetry relates to the spatial extension of each region and the value of the potential derivative in these regions.

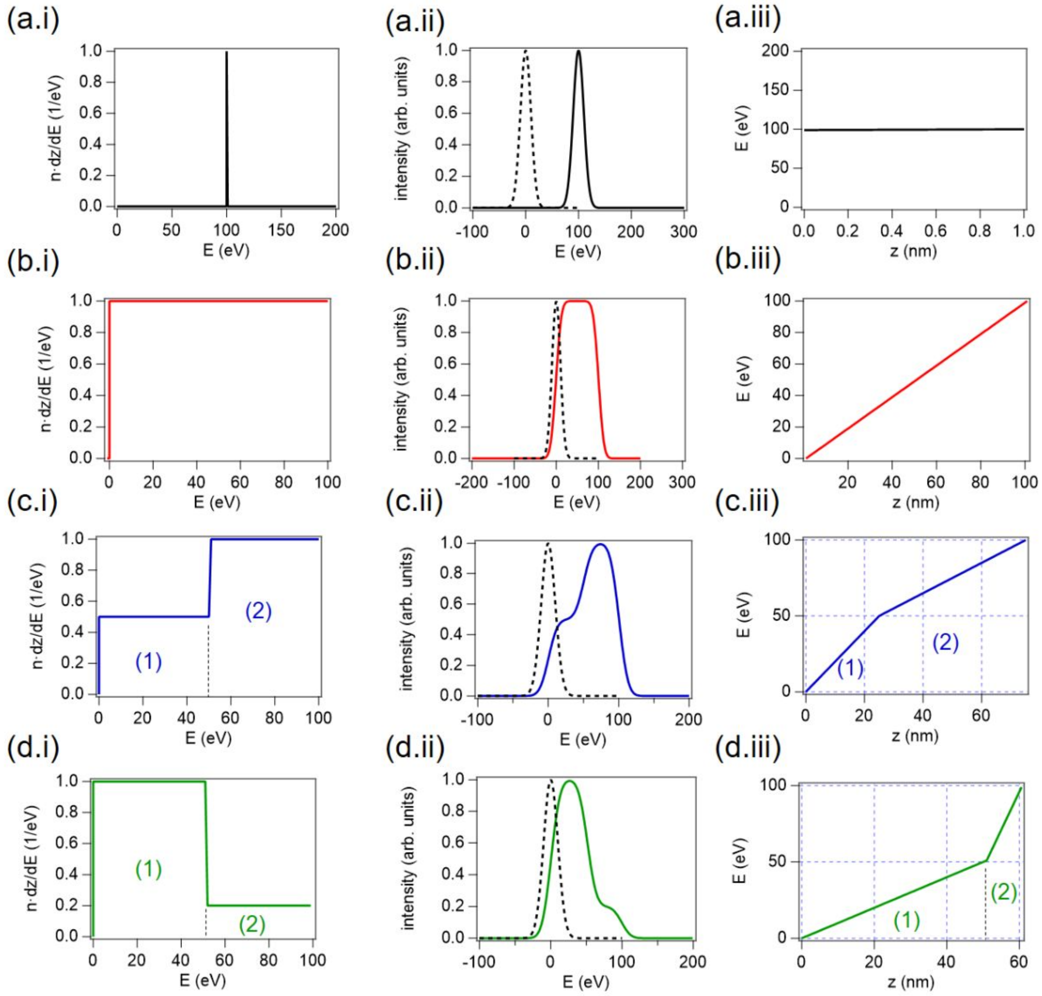

Fig. S33: Effect of convolution operation on a Gaussian function (ii) with a function (i) representing the inverse gradient of the potential and the respective real potential-space diagram (iii), given for four different cases: a spatially constant potential with value V=100 (a), a spatially linearly increasing potential (b), a spatially linearly increasing potential with a reduction of factor 2 of the slope after a third of the spatial extension (c), a spatially linearly increasing potential with a doubling of the slope after four-fifth of the spatial extension (e).

After this first explanatory description of the relation of the convolution operation and electronic states in spatially varying potential landscapes, in the following, the procedure that allowed the reconstruction of the potential landscape is shown.

Fig. S34 shows how the potential landscape over the entire BL-MoS_2/HOPG heterostructure upon deposition of 0.5 Å of the p-type acceptor molecule F_6TCNNQ was found. In subFig.(a) the step function allowing to reproduce the EDC around Γ is shown. This function gives the corresponding potential curve shown in Fig. S34(b) by the blue circled markers. This potential curve describes well the found values of the core level shifts of molybdenum. As no information of the potential landscape outside the TMDC spatial extent (0.0 - 13.2) Å can be accessed by the MoS_2 core or valence levels, the potential landscape outside of this range had to be extrapolated, knowing that the surface value will correspond to the work function change (of 0.55 eV found for this specific deposition of the molecular acceptor) while the potential at the HOPG surface should go down to 0 eV. The results of these procedures are shown by the blue solid line. Concomitantly, it shows a constant potential within the HOPG substrate after a small decay caused by the increased hole density in the first atomic layers, and considers the constraints of the boundary conditions at the molecule-inorganic and inorganic-semimetal interface. In order to remove the non-differentiable points of the potential, which will cause abrupt kinks in second derivative corresponding to the differential charge density, a smoothing procedure was applied to the potential curve. The results are shown by the solid red line. As strong kinks and singularities in the charge or potential distribution are unphysical, the smoothed curve represents more likely the actual electronic properties of the sample. Fig. S34(c) shows again the potentials, allowing the direct comparison between the potential in the initially smoothed version and the final potential landscape shown in the main paper. This last potential gives the optimized differential charge distribution (solid black line in Fig. S34(d)), not featuring the small wiggles.

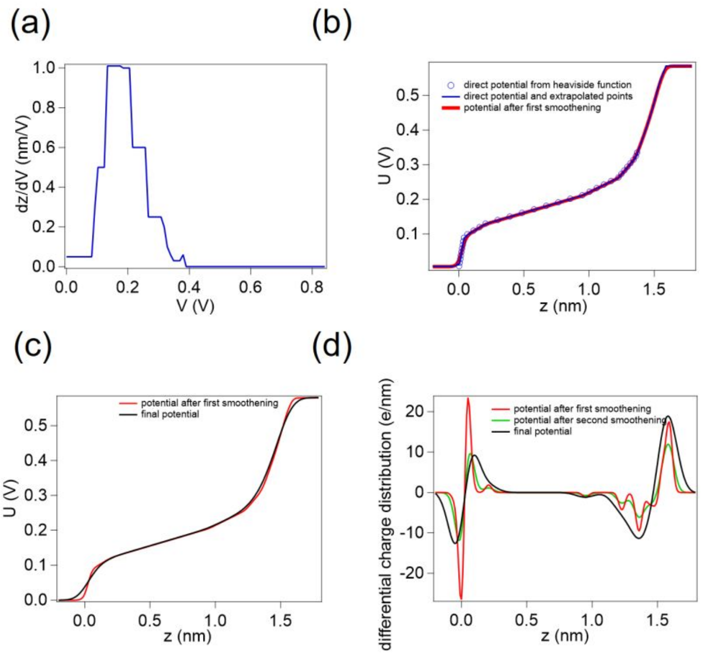

Fig. S34: Extraction of final potential landscape in the case of a deposition of 0.5 Å of the molecular p-type donor F_6TCNNQ on the BL-MoS_2 on HOPG. SubFig. (a) shows the Heaviside function, displaying the inverse potential-space gradient over the potential found. In subFig. (b) the potential landscape found by direct integration of the Heaviside function over the spatial range of the TMDC-BL (0-13.2) Å and inversion is shown, as well as the later extrapolated points of the potential in the interface regions of the TMDC with the organic molecule and the substrate, prior and upon a smoothening procedure. SubFig. (c) shows the evolution of the potential through different smoothening procedures and the final potential found by the optimized differential charge distributions which are shown in (d) and are found by differentiating the potential over space.

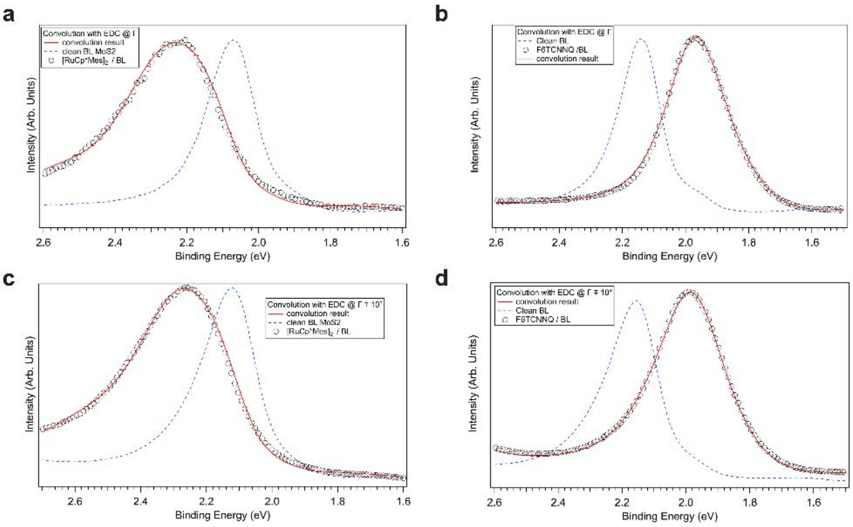

Figure S35: None integrated (±0.1°) (a,b) and ±10° integrated (c,d) EDCs at Γ before and after deposition of and [RuCp*mes]$_2$ (a,c) and F_6TCNNQ (b,d) to highlight the change in lineshape induced by molecular adsorption. Each subfigure shows the as-measured clean and either F_6TCNNQ or [RuCp*mes]$_2$ EDCs together with the simulated EDC obtained by convoluting the clean BL EDC with a function representing the potential gradient affecting the BL-MoS_2 orbitals.

Fig. S35. show an overlay of the EDC of clean BL-MoS_2 with the EDCs of BL-MoS_2 covered with F_6TCNNQ and [RuCp*mes]$_2$ with and without an angle integration of ±10°. Note here, that the integration over a larger k// region, as for the integration of ±10°, shifts the peak maximum of the quantum well states towards higher BE because of the spectral weight at higher energies for higher k//. In the main manuscript only the momentum-integrated (±10° around Γ) EDCs are presented. This was reasoned as the integration procedure minimized the possible impact of scattering effects of MoS_2 photoelectrons by the molecular layer. Here we show that the convolution results are equal for both cases, as can be clearly seen shown in Fig. S35. Therefore the reasoning given in the manuscript, is adequate also for other integration regions.

10. Simulation of core level data: convolution approach

Analogously to the reconstruction of the VB states by the convolution operation of the inverse space differentiated potential gradient, also a reconstruction of the core levels must be possible in the same technique. This allows testing once more the adequacy of the technique and to check whether the found potential is in line with the real potential landscape within the sample.

The results of the convolution of the clean BL S 2p core levels with the gradient function of the found potentials upon molecular donor and acceptor deposition of 1.6 Å and 0.5 Å, respectively, is shown in Fig. S36. As can be clearly seen, the binding energy shift as well as line broadening is reproduced within the experimental uncertainty of 30 meV for both molecular depositions.

When the two core levels are superimposed to one another, the asymmetrical broadening of the core levels upon molecular deposition, for both dopants, can be clearly observed. This means, that in

general by simply using the XPS data, a complete reconstruction of the potential profile within the BL-MoS_2 should be accessible.

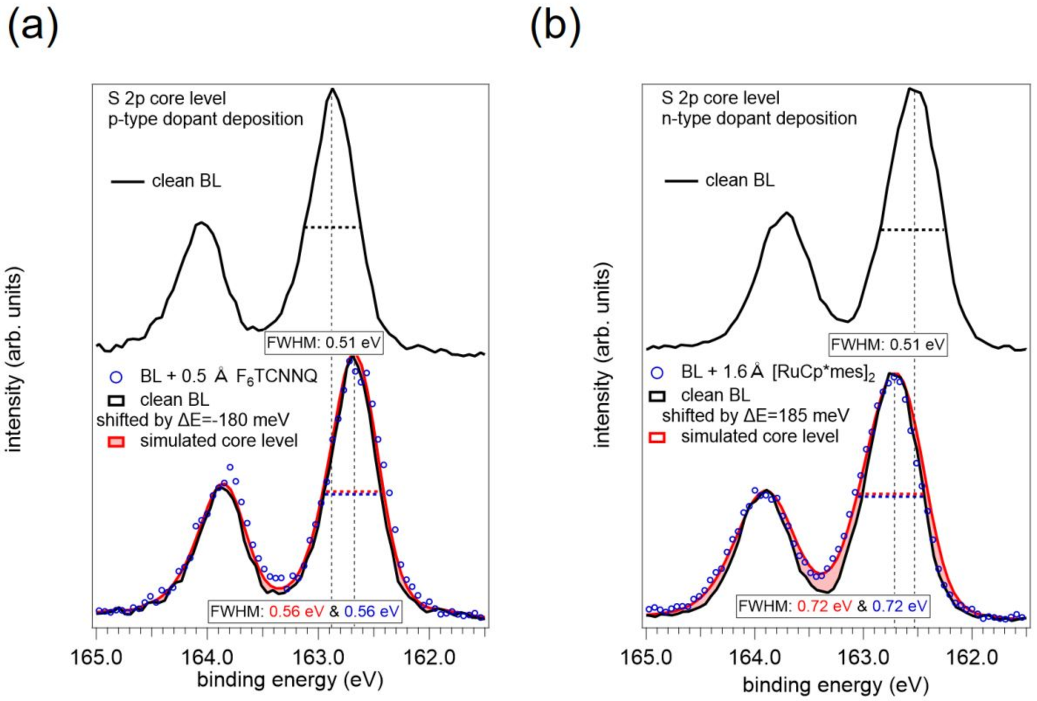

Fig. S36: Results of reconstruction of the experimentally found core level peak S 2p by convolution function given by the inverse gradient of the potential defined for a deposition of 0.5 Å of the molecular p-type dopant F_6TCNNQ (subFig. (a)) and 1.6 Å of the molecular n-type dopant $[RuCp^*mes]_2$ (subFig. (b)).

11. *Renormalization of the as-determined potential by the attenuation length from inelastic mean free path*

Here we renormalized the magnitude of the convolution function used to track the potential in the BL-MoS_2 after deposition of F_6TCNNQ by a (continuous) exponential function representing the attenuation and then proceed with obtaining the potential as explained above. We see that the effect of the attenuation only brings minor changes to the results which are probably within the uncertainty of the method.

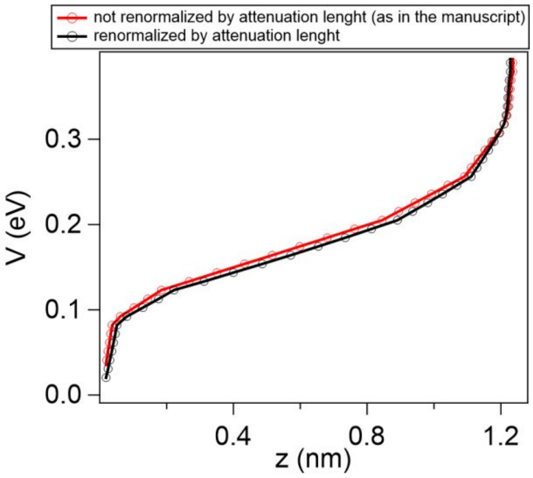

Fig. S37: comparison between as-determined potential from EDC and renormalized potential including attenuation due to IMFP.

12. *Quantum confined Stark effect and Eigenenergies at Γ-point*

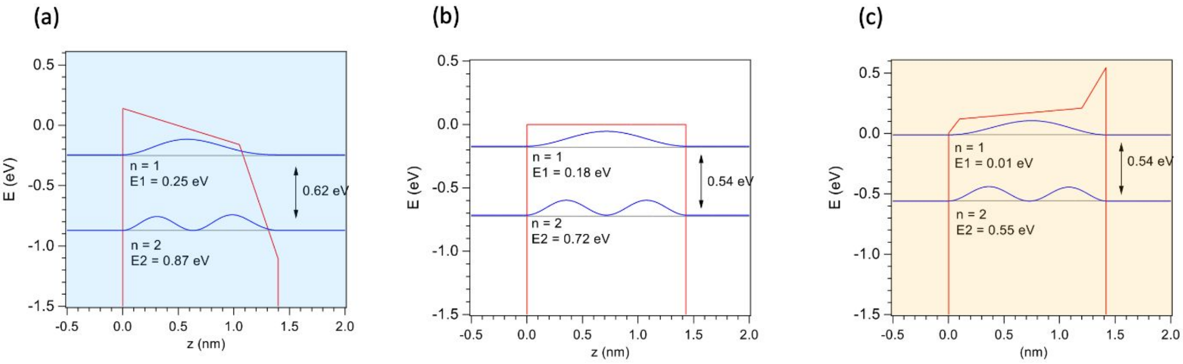

Fig. S38: Potential well, Eigenenergies and probability distribution of the QW states for the (a) [RuCp*mes]$_2$ / BL-MoS_2, (b) clean BL-MoS_2 and (c) F_6TCNNQ / BL-MoS_2 heterojunctions.

Understanding the quantum-confined Stark effect (QCSE) in these low-dimensional systems is fundamental for a full comprehension of their optoelectronic properties. Here, we finally investigate these heterostructures from a quantum-mechanical perspective by modeling the systems as finite potential wells (where the well represents the BL-MoS_2) with potential gradients consisting of those actually depicted in Fig. 7.c and 7.f. For the clean BL-MoS_2, the width (W)of the QW is 14.3 Å, which overestimates slightly the actual width because of the infinite height of the well. An effective mass of 1 m_0 is used. The experimental energy difference between the n1 and n2 QWS $\Delta E_{exp}^{\Gamma_2-\Gamma_1}$ of 0.530 eV ± 5 meV between Γ_1 and Γ_2 is fully reproduced by the theoretical energy difference $\Delta E_{th}^{n_2-n_1}$ of 0.540 eV between the n_1 and n_2 states.

Fig. S38.c shows the potential well portraying the case of the F_6TCNNQ / BL-MoS_2 / HOPG junction. The applied potential derived from the previous analysis results in an energy shift of the n_2 state by 170 ± 5 meV to higher energy, which compares almost perfectly with the 174 ± 5 meV experimentally observed. $\Delta E_{th}^{n_2-n_1}$ is basically constant within 5 meV, which is in the range of the measured $\Delta E_{exp}^{\Gamma_2-\Gamma_1}$ of ca 7 meV ± 5 meV.

The potential well simulating the case of $[RuCp^*mes]_2$ is depicted in Fig. S38.a. It shows an energy shift of Γ_2 by -150 ± 5 meV. Here again, we find an excellent agreement with the experimental change of -143 ± 5 meV. $\Delta E_{exp}^{\Gamma_2-\Gamma_1}$ is also seen to increase substantially by 75 ± 5 meV for this case. This is well-reproduced in the calculations by slightly narrowing the width of the well to 1.40 Å, yielding $\Delta E_{th}^{\Gamma_2-\Gamma_1}$ of 80 ± 5 meV. With a width of 1.43 Å, the $\Delta E_{th}^{\Gamma_2-\Gamma_1}$ is underestimated by 20 meV. Such a change in the width of the potential well may reflect further electronic confinement upon adsorption of the $[RuCp^*mes]_2$, either stemming from a narrower d_{z^2} orbital redistribution in relation to the charging of the CBM or to a decrease in the (likely corrugated) MoS_2 interlayer distance[13–15].

Bibliography

1. Splendiani, A. *et al.* Emerging photoluminescence in monolayer MoS2. *Nano Lett.* **10**, 1271–1275 (2010).

2. Xiao, D., Liu, G. Bin, Feng, W., Xu, X. & Yao, W. Coupled spin and valley physics in monolayers of MoS 2 and other group-VI dichalcogenides. *Phys. Rev. Lett.* **108**, 196802 (2012).

3. Brumme, T., Calandra, M. & Mauri, F. First-principles theory of field-effect doping in transition-metal dichalcogenides: Structural properties, electronic structure, Hall coefficient, and electrical conductivity. *Phys. Rev. B - Condens. Matter Mater. Phys.* **91**, 155436 (2015).

4. Pierucci, D. *et al.* Large area molybdenum disulphide- epitaxial graphene vertical Van der Waals heterostructures. *Sci. Reports* **6**, 1–10 (2016).

5. Molina-Sánchez, A. & Wirtz, L. Phonons in single and few-layer MoS 2 and WS 2. *Phys. Rev. B* **84**, 155413 (2011).

6. Lee, K. Y. *et al.* Raman imaging of strained bubbles and their effects on charge doping in monolayer WS2 encapsulated with hexagonal boron nitride. *Appl. Surf. Sci.* **604**, 154489 (2022).

7. Madapu, K. K. & Dhara, S. Laser-induced anharmonicity vs thermally induced biaxial compressive strain in mono- And bilayer MoS2grown via CVD. *AIP Adv.* **10**, (2020).

8. Ng, L. W. T. *et al.* Printing of graphene and related 2D materials: Technology, formulation and applications. *Print. Graphene Relat. 2D Mater. Technol. Formul. Appl.* 1–216 (2018).

9. Wurstbauer, U., Miller, B., Parzinger, E. & Holleitner, A. W. Light–matter interaction in transition metal dichalcogenides and their heterostructures. *J. Phys. D. Appl. Phys.* **50**, 173001 (2017).

10. S. Guo, S. B. Kim, S. K. Mohapatra, Y. Qi, T. Sajoto, A. Kahn, S. R. Marder, S. Barlow, n-Doping of Organic Electronic Materials using Air-Stable Organometallics, *Adv. Mater.* **24**, 699 (2012).

11. Zhang, F., Kahn, A., Investigation of the High Electron Affinity Molecular Dopant F6-TCNNQ for Hole-Transport Materials. *Adv. Funct. Mater.* **28**, 1703780 (2018).

12. Cheng, Y. & Schwingenschlögl, U. MoS2: A First-Principles Perspective. In: *MoS2. Lecture Notes in Nanoscale Science and Technology*; Wang, Z., Eds.; Springer, Cham: 2014; pp 103-128.

13. Jones, A. J. H. *et al.* Visualizing band structure hybridization and superlattice effects in twisted MoS2/WS2 heterobilayers. *2D Mater.* **9**, 015032 (2021).

14. Xiao, J. *et al.* Effects of van der Waals interaction and electric field on the electronic structure of bilayer MoS2. *J. Phys. Condens. Matter* **26**, 405302 (2014).

15. Arnold, F. M., Ghasemifard, A., Kuc, A., Kunstmann, J. & Heine, T. Relaxation effects in twisted bilayer molybdenum disulfide: structure, stability, and electronic properties. *2D Mater.* **10**, 045010 (2023).
